# Three new *Pristimantis* species in the subgenus *Huicundomantis* (Amphibia: Anura: Strabomantidae) from Reserva Biológica Cerro Plateado, southern Ecuador

**DOI:** 10.7717/peerj.20930

**Published:** 2026-03-11

**Authors:** Paul Székely, Santiago Hualpa-Vega, Daniel Hualpa-Vega, Diana Székely

**Affiliations:** 1 Museo de Zoología, Universidad Técnica Particular de Loja, Loja, Ecuador; 2 Laboratorio de Ecología Tropical y Servicios Ecosistémicos (EcoSs-Lab), Facultad de Ciencias Exactas y Naturales, Departamento de Ciencias Biológicas y Agropecuarias, Universidad Técnica Particular de Loja, Loja, Ecuador; 3 Faculty of Natural and Agricultural Sciences, Ovidius University Constanța, Constanța, Romania; 4 Fundación Green Jewel, Loja, Ecuador

**Keywords:** Habitat specialization, Gold mining, Phylogenetics, *Pristimantis nangaritza*, Rain frogs, Sandstone plateau, Systematics, Tropical Andes

## Abstract

We describe three new species of direct-developing frogs of the genus *Pristimantis*, subgenus *Huicundomantis*, from the Reserva Biológica Cerro Plateado in southern Ecuador, one of the most biodiverse yet least explored regions of the Cordillera del Cóndor. Combining molecular, morphological, and bioacoustic data, we provide diagnoses and phylogenetic placements for *Pristimantis verrucosus* sp. nov., *P. plateado* sp. nov., and *P. melanops* sp. nov. The first two species are placed within the *P. cryptomelas* species group, while the third belongs to the *P. phoxocephalus* group. The new species can be readily distinguished from their local congeners by the following combination of traits: *P. verrucosus* sp. nov. exhibits a coarsely tuberculate dorsum and flanks, lacks dermal folds or ridges in the occipital/scapular region, has a distinct tympanic annulus and membrane, low cranial crests, Toe V longer than Toe III, and males possess a vocal sac and slits; *P. plateado* sp. nov. has a silver or light bronze iris, lacks dermal folds or ridges in the occipital/scapular region, a rounded snout in dorsal and lateral views, a distinct tympanic annulus and membrane, Toe V longer than Toe III, and males lack a vocal sac and slits; *P. melanops* sp. nov. is characterized by a uniquely blackish iris with a white circumpupillary ring and coarsely pustulate flanks. Additionally, we provide the first description of the advertisement call for a species in the *P. cryptomelas* group, thereby contributing novel bioacoustic data for a clade in which vocalizations were previously unknown. All three species are endemic to a single locality and two exhibit ecological specialization, being bromeliad specialists. Given their extremely restricted distributions and the ongoing threat of habitat degradation from illegal mining activities close to their distribution site, we recommend that all three species be listed as Critically Endangered under IUCN criteria.

## Introduction

The Tropical Andes are one of the world’s most species-rich regions and a hotspot of biodiversity (Myers et al., 2000), leading in terms of number of endemic species (Mittermeier et al., 2011). Additionally, they are estimated to still hold large numbers of species currently unknown to science (Moura & Jetz, 2021). The lack of accurate information regarding existing species, their distribution and ecology, is one of the greatest challenges to effective conservation planning, making the Tropical Andes a key region for concentration of research efforts.

A distinctive part of the Tropical Andes is the Cordillera del Cóndor, a relatively isolated mountain range shared by Ecuador and Peru. Due to its unique geology and climate, the area has been regarded as a center of endemism, likely to have the highest concentration of undescribed plant species in the world (Neill, 2007). The Reserva Biológica Cerro Plateado (RBCP) is one of the newest protected areas from Cordillera del Condor, located in the province of Zamora Chinchipe, southern Ecuador. Established in 2010, the RBCP spans approximately 30,760 hectares, with elevations ranging from 840 to 3,120 m above sea level (Ministerio del Ambiente del Ecuador, 2018). The reserve’s sandstone plateaus, which exhibit tepui-like features, share geological and ecological affinities with formations of the Guiana Shield, supporting isolated habitats that foster high levels of endemism and speciation (Neill, 2005; Jadán & Mendoza, 2013). Despite its incredible biodiversity, the RBCP remains one of the least explored areas in this region, few studies having been carried out here (Valencia et al., 2023).

A joint team of researchers from Universidad Estatal Amazónica, Escuela Politécnica Nacional and the NGO Naturaleza y Cultura Internacional was the first to conduct a scientific expedition in the reserve in August 2012 (Almendáriz et al., 2014). A second expedition was carried out in September 2016 by a team from Museo de Zoología, Pontificia Universidad Católica del Ecuador. As a result of these expeditions, two frog species were described from RBCP: the charismatic hylid *Hyloscirtus condor* Almendáriz et al., 2014 and the bromeliad specialist rain frog *Pristimantis muranunka* Brito et al., 2017. Since November 2018, a research team from the Museo de Zoología, Universidad Técnica Particular de Loja has been conducting fieldwork in the reserve and its buffer zone, extending their surveys into previously unsampled areas. Based on all available data, approximately 60 amphibian species have been reported from the RBCP, representing three orders and eleven families (Hualpa-Vega, 2020). Of these, 17 are new, currently undescribed (candidate) species, including 12 frogs belonging to the genus *Pristimantis* Jiménez de la Espada, 1870.

Among vertebrates, amphibians are the most threatened (IUCN, 2025), but also show the highest recent rates of species description (Liu et al., 2022). The Neotropical genus of direct-developing frogs *Pristimantis* (Amphibia: Anura: Strabomantidae) is the most species-rich vertebrate genus, currently comprising 626 described species (Frost, 2025), with many additional species awaiting formal description. The genus is distributed across Central and South America, with the highest species diversity concentrated in the Andean regions of Colombia, Ecuador, and Peru (Kieswetter & Schneider, 2013). The need to organize and classify the rapidly increasing number of *Pristimantis* species became apparent as early as the 1970s, when Lynch (1976) proposed a series of species groups for the South American representatives of the genus, which at that time was classified under *Eleutherodactylus*. Although species groups are informal classifications, they serve as an essential tool for managing highly diverse genera such as *Pristimantis*. By organizing species according to shared genetic, morphological, or ecological traits, these groups facilitate taxonomic discussions and provide a framework for describing new species.

Several of these species groups have been incorporated into subgenera to better organize the species within this highly diverse genus into well-supported clades, a trend that has intensified in recent years (Páez & Ron, 2019; Franco-Mena et al., 2023; Yánez-Muñoz et al., 2025). In southern Ecuador, one of the most representative subgenera is *Huicundomantis* (Páez & Ron, 2019), which currently comprises three species groups: the *Pristimantis miktos* group (three species), the *P. cryptomelas* group (six species), and the *P. phoxocephalus* group (23 species), along with two species unassigned to any group and seven undescribed candidate species (Székely et al., 2026; Fig. S1).

Herein, we describe three species from the subgenus *Huicundomantis* using an integrative taxonomy approach that combines molecular, morphological, and bioacoustic data.

## Materials AND methods

### Ethics statement

This study was carried out in compliance with the guidelines for use of live amphibians and reptiles in field research compiled by the American Society of Ichthyologists and Herpetologists, the Herpetologists’ League and the Society for the Study of Amphibians and Reptiles. Research permits were issued by the Ecuadorian Ministry of Environment (MAE-DNB-CM-2015-0016 and MAATE-ARSFC-2024-1019).

### Nomenclatural acts

The electronic version of this article in Portable Document Format (PDF) will represent a published work according to the International Commission on Zoological Nomenclature (ICZN), and hence the new names contained in the electronic version are effectively published under that Code from the electronic edition alone. This published work and the nomenclatural acts it contains have been registered in ZooBank, the online registration system for the ICZN. The ZooBank LSIDs (Life Science Identifiers) can be resolved and the associated information viewed through any standard web browser by appending the LSID to the prefix http://zoobank.org/. The LSID for this publication is: urn:lsid:zoobank.org:pub:DD6ADBA4-1D9C-4992-B44B-1AF41B091196. The online version of this work is archived and available from the following digital repositories: PeerJ, PubMed Central SCIE and CLOCKSS.

### Specimen collection and study site

Fieldwork consisted of several expeditions conducted between November 2018 and September 2025 in the Reserva Biológica Cerro Plateado (Zamora Chinchipe Province, southern Ecuador; 4.6349°S, 78.8902°W; datum WGS84; 2,099 m a.s.l.). We carried out intensive visual encounter and auditory surveys both during the day and at night. Habitats are defined according to the Ecosystem Classification System of Continental Ecuador (Ministry of the Environment of Ecuador, 2013). We refer to a locality as a geographically or ecologically distinct area in which a single threatening event can rapidly affect all occurrences of an ecosystem type (IUCN, 2024).

All collected specimens were photographed alive, euthanized with 20% benzocaine, fixed in 10% formalin, and stored in 70% ethanol; tissue samples for genetic analyses were preserved in 96% ethanol. Examined specimens (listed in the type-series and Appendix 1) were deposited at the Museo de Zoología, Universidad Técnica Particular de Loja, Loja, Ecuador (MUTPL).

### Molecular analyses

Genomic extraction, amplification, and sequencing were as described in Székely et al. (2020); briefly, from liver tissue preserved in 96% ethanol, we extracted DNA and amplified it through PCRs using Extract-N-Amp™ Tissue PCR Kit (Sigma-Aldrich, Merck KGaA, Darmstadt, Germany) following the manufacturer’s protocol. PCR products were purified by PEG precipitation and sequenced at Macrogen Sequencing Service (Macrogen Inc., Seoul, Korea). The newly generated DNA sequences were deposited in GenBank (Appendix 2). For the phylogenetic analysis we used sequences of two mitochondrial ribosomal genes (*12S* and *16S* rRNA) and one nuclear gene (recombination-activating gene 1, *RAG-1*) from 79 specimens of 37 species and seven candidate species corresponding to 42 different localities from Ecuador and two from Peru (Appendix 2); these samples (GenBank-available sequences and 21 new sequences generated by this study) represent all the currently confirmed species of the *Huicundomantis* subgenus of *Pristimantis* (*sensu* Ortega, Brito & Ron, 2022). As outgroups, we used *Pristimantis unistrigatus* (Günther, 1859), *P. ceuthospilus* (Duellman & Wild, 1993), *P. imitatrix* (Duellman, 1978), *P. diadematus* (Jiménez de la Espada, 1875), *P. rhodoplichus* (Duellman & Wild, 1993), *P. melanogaster* (Duellman & Pramuk, 1999), *P. wiensi* (Duellman & Wild, 1993), *P. simonsii* (Boulenger, 1900), *P. orestes* (Lynch, 1979), *P. colodactylus* (Lynch, 1979) and *P. orcesi* (Lynch, 1972), and the tree was rooted with *P. unistrigatus*.

The sequences were edited, assembled and aligned (MAFFT algorithm with the G-INS-i iterative refinement method; Katoh & Standley, 2013) in Geneious Prime (v2025.2.2; Biomatters Ltd., Auckland, New Zealand). The aligned sequences were visually inspected to correct alignment errors and concatenated into a single matrix in PhyDE (Müller et al., 2010), then used for the phylogenetic analyses. The analyses were based on a 2,424-base pair (bp) dataset (923 bp for *12S*, 889 bp for *16S* and 612 bp for *RAG-1*). The aligned and concatenated matrix is available at https://doi.org/10.5281/zenodo.17329629.

Phylogenetic relationships were inferred using both Maximum Likelihood (ML) and Bayesian Inference (BI). We used PartitionFinder v. 2.1.1 (Lanfear et al., 2017) to select the best partition scheme with the Bayesian Information Criterion (BIC) as a model of selection; Partition-Finder identified three partition schemes (best model in parentheses): *12S* and *16S* (GTR+I+G), *RAG-1* 1st position (K80+G), and *RAG-1* 2nd and 3rd position (F81+I+G). The ML analyses were conducted in IQ-TREE 2 (Minh et al., 2020). We performed two different runs, in order to test the tree’s topologies. One was carried out with IQ-TREE’s default settings, in which the program determined the best-fit model for our alignment (TIM2+F+I+R3 under BIC), and with the -bnni option (optimized, hill-climbing nearest neighbor interchange, NNI, search) in order to reduce the risk of overestimating the branch supports of the UFBoot, with 100,000 bootstrap replicates for the SH-like approximate likelihood ratio branch test (SH-aLRT; Guindon et al., 2010) and with 100,000 ultrafast bootstrap replicates (UFBoot; Hoang et al., 2018) to assess the branch support. The second run was performed with the three partition schemes identified by Partition-Finder and branch support evaluated with 1,000 standard non-parametric bootstrap (Boot; Felsenstein, 1985) searches. The BI analysis was implemented in MrBayes v.3.2.6 (Ronquist et al., 2012) using the partition schemes identified by Partition-Finder. The Markov Chain Monte Carlo (MCMC) runs were performed twice, independently, for 10 million generations, with trees sampled every 1,000 generations. Convergence of the runs was assessed from the average split frequency of standard deviations (*p* < 0.001) and by checking the potential scale reduction factors (PSRF ~1.0) for all model parameters. Consensus trees were summarized after discarding the initial 25% as burn-in (Székely et al., 2023).

We *a priori* regarded that a tree node had “strong support” when its bootstrap (Boot) value was >75 and its Bayesian posterior probability (PP) was >0.95, “moderate support” for 50–75 and 0.90–0.95, and “weak support” or non-resolved for values lower than 50 and 0.90, respectively (Székely et al., 2023); clades are considered strongly supported when UFBoot ≥95 and SH-aLRT ≥80 (Guindon et al., 2010; Hoang et al., 2018). Uncorrected genetic p-distances were calculated using a trimmed 862 bp fragment of the *16S*, excluding regions with extensive gaps. The analysis was performed in MEGA6 (Tamura et al., 2013) using the pairwise deletion option. The results are presented in File S1.

### Morphological analyses

We mostly follow the definitions provided by Lynch & Duellman (1997) and Ospina-Sarria & Duellman (2019), and the format of the description proposed by Duellman & Lehr (2009). Sex was determined by gonadal inspection and the presence of secondary sexual characters like the vocal sacs and slits in males. Coloration of live specimens was based on digital photographs.

All adult specimens were weighed (Body Mass: BM) before euthanasia using a My Weigh Triton T3 portable scale with 0.01 g precision. Specimens were measured by the same person (PS) under a stereo microscope, with a Vernier caliper, and values were rounded to the nearest 0.1 mm. The following morphometric variables were measured: (1) Snout-vent length (SVL), distance from the tip of snout to the posterior margin of vent; (2) head width (HW), widest portion of the head, measured at the level of jaw articulation; (3) head length (HL), distance from the tip of snout to the posterior angle of jaw articulation; (4) interorbital distance (IOD), the shortest distance between the inner margins of the orbits; (5) internarial distance (IND), distance between the inner edges of the narial openings; (6) upper eyelid width (EW), greatest width of the upper eyelid margins; (7) eye diameter (ED), horizontal distance between the anterior and posterior corners of the eye; (8) eye-nostril distance (EN), distance from the posterior margin of the nostril to the anterior corner of the eye; (9) tympanum diameter (TD), greatest horizontal distance between the peripheral borders of tympanic annulus; (10) thigh length (THL), length of thigh from vent to knee; (11) tibia length (TL), length of flexed leg from knee to heel; (12) foot length (FL), distance from proximal margin of inner metatarsal tubercle to tip of Toe IV; (13) forearm length (FLL), length of forearm from the flexed elbow to proximal edge of palmar tubercle; (14) hand length (HAL), distance from proximal edge of palmar tubercle to tip of Finger III. Measurements are given as mean ± SD.

To ensure that the newly described species were indeed undescribed and not previously named taxa lacking genetic data, we conducted a comprehensive morphological comparison. Specifically, we examined diagnostic traits of our specimens against all described species of *Pristimantis* known from Ecuador and northern Peru. This comparison focused particularly on distinctive qualitative characters such as skin texture, tuberculation, cranial crests, folds, finger and toe shapes, and iris coloration, among others. Reference data were obtained from original species descriptions, taxonomic revisions, and relevant literature. By integrating these morphological assessments with molecular and bioacoustic evidence, we confirmed that the species presented herein are distinct from all currently recognized congeners.

### Bioacoustics analyses

The calls were recorded in the field using an Olympus LS-11 Linear PCM Recorder, with a RØDE NTG2 condenser shotgun microphone, at 44.1 kHz sampling frequency and 16-bit resolution, in WAV file format. Air temperature and humidity were measured with a data logger (model EL-USB-2-LCD; Lascar Electronics, Erie, PA, USA, accuracy: ± 0.5 °C; ± 5%). All analyzed call recordings are deposited in their original form, full length at Fonoteca UTPL (record IDs are provided in the File S2). Acoustic analysis was conducted using Raven Pro 1.6 (K. Lisa Yang Center for Conservation Bioacoustics at the Cornell Lab of Ornithology). We measured the temporal parameters from oscillograms and the spectral parameters from spectrograms obtained with the Hanning window function, DFT: 512 samples, 3 dB filter bandwidth: 124 Hz, and a 50% overlap (Székely et al., 2020).

The terminology and procedures for measuring call parameters follow Cocroft & Ryan (1995), Toledo et al. (2015), and Köhler et al. (2017), with a note-centered approach to distinguish between a call and a note (*sensu* Köhler et al., 2017). The following temporal and spectral parameters were measured and analyzed: (1) *call duration*: time from the beginning to the end of a call (for multi-note calls); in the case of single-note calls, this is the same as a note duration; (2) *inter-call interval*: the interval between two consecutive calls, measured from the end of one call to the beginning of the consecutive call; (3) *call rate*: number of calls/minute, measured as the time between the beginning of the first call and the beginning of the last call; (4) *note duration*: the duration of a single note within a call, measured from beginning to the end of the note; (5) *inter-note interval*: the interval between two consecutive notes within the same call, measured from the end of one note to the beginning of the consecutive note; (6) *note rate*: number of notes per second, measured as the time between the beginning of the first note and the beginning of the last note; (7) *dominant frequency*: the frequency containing the highest sound energy, measured along the entire call; and (8) *the 90% bandwidth*: reported as frequency 5% and frequency 95%, or the minimum and maximum frequencies, excluding the 5% below and above the total energy in the selected call (Székely et al., 2020). Measurements are given as mean ± SD.

## Results

### Phylogenetic analyses

**Figure 1:**
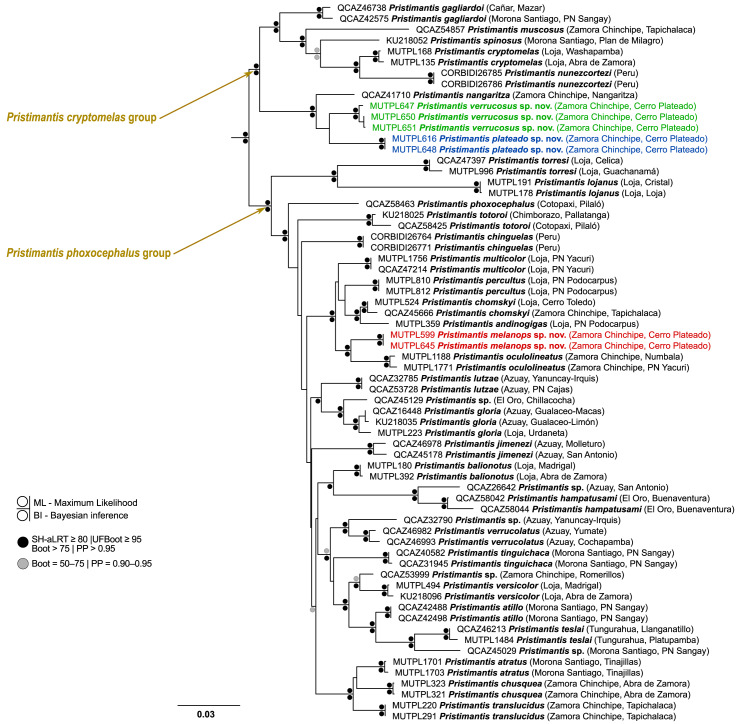
Maximum likelihood phylogram of the *Pristimantis cryptomelas* and *P. phoxocephalus* species groups. The tree is based on a 2,424-base pair dataset of concatenated mitochondrial DNA from *12S* and *16S*, and nuclear DNA from *RAG-1* gene fragments. Outgroup is not shown; the tree was rooted with *P. unistrigatus* (the complete tree of the *Pristimantis* (*Huicundomantis*) subgenus is provided in the Fig. S1). The catalog number, species name, province and short locality name of the vouchers are shown next to each terminal; samples are from Ecuador and Peru (associated data are listed in Appendix 2). Abbreviations: SH-aLRT = SH-like approximate likelihood ratio branch test, UFBoot = ultrafast bootstrap, Boot = standard non-parametric bootstrap, PP = Bayesian posterior probabilities.

The Bayesian Inference and the two Maximum Likelihood phylogenetic trees showed very similar topologies, with only minor differences in the position of some of the unresolved branches. The BI tree presented relatively higher branch support, while in the case of ML, the UFBoot tree was the most conservative (having overall smaller support values) compared to the non-parametric Boot tree (Fig. 1). We recovered the subgenus *Huicundomantis* as monophyletic, with strong support (SH-aLRT = 97.9%; UFBoot = 99%; Boot = 97%; PP = 1), in accordance with Páez & Ron (2019), Ortega, Brito & Ron (2022), Sánchez-Nivicela et al. (2024) and Székely et al. (2026). However, as shown in our phylogenetic tree (Fig. S1), and similarly to Sánchez-Nivicela et al. (2024) and Székely et al. (2026), the basal terminals within *Huicundomantis* are represented by the strongly supported clade comprising *P. philipi* (Lynch & Duellman, 1995) and *P. ruidus* (Lynch, 1979), rather than the *P. miktos* group. Nonetheless, this topology remains unresolved due to the lack of sufficient branch support (Boot = 70%; Fig. S1).

Our results indicate that two of the new species fall within the *Pristimantis cryptomelas* species group (Fig. 1), are sister taxa to *P. nangaritza* Páez & Ron, 2019, and together form a strongly supported clade (SH-aLRT = 99.4%; UFBoot = 100%; Boot = 100%; PP = 1). The third species is recovered as the sister taxon to *P. oculolineatus* Székely et al., 2026, a morphologically similar species to *P. atratus* (Lynch, 1979); together with *P. multicolor* Páez & Ron, 2019, *P. percultus* (Lynch, 1979), *P. chomskyi* Páez & Ron, 2019, and *P. andinogigas* Yánez-Muñoz et al., 2019, they form a strongly supported clade (SH-aLRT = 99.9%; UFBoot = 100%; Boot = 99%; PP = 1) in the *P. phoxocephalus* species group (Fig. 1).

Uncorrected genetic p-distances for the gene *16S* between *P. nangaritza* and its sister species (*P. verrucosus* sp. nov. and *P. plateado* sp. nov.) range between 3.4% and 4.1%; the distances between the two new species range between 3.7% and 3.9%; and the distances from the other species of the *P. cryptomelas* group range between 5.8% and 8.9% (File S1). The genetic p-distances for *P. melanops* sp. nov. and its sister species *P. oculolineatus* range between 3.6% and 3.8%; the distances from the other species of their clade range between 3.7% and 5.2%; and the distances from the other species of the *P. phoxocephalus* group range between 4.8% and 11.6% (File S1). As for the intraspecific genetic p-distances, these did not differ among individuals within any of the three new species (File S1).

### Systematics

Family Strabomantidae Hedges, Duellman & Heinicke, 2008

Genus *Pristimantis* Jiménez de la Espada, 1870

**Figure 2:**
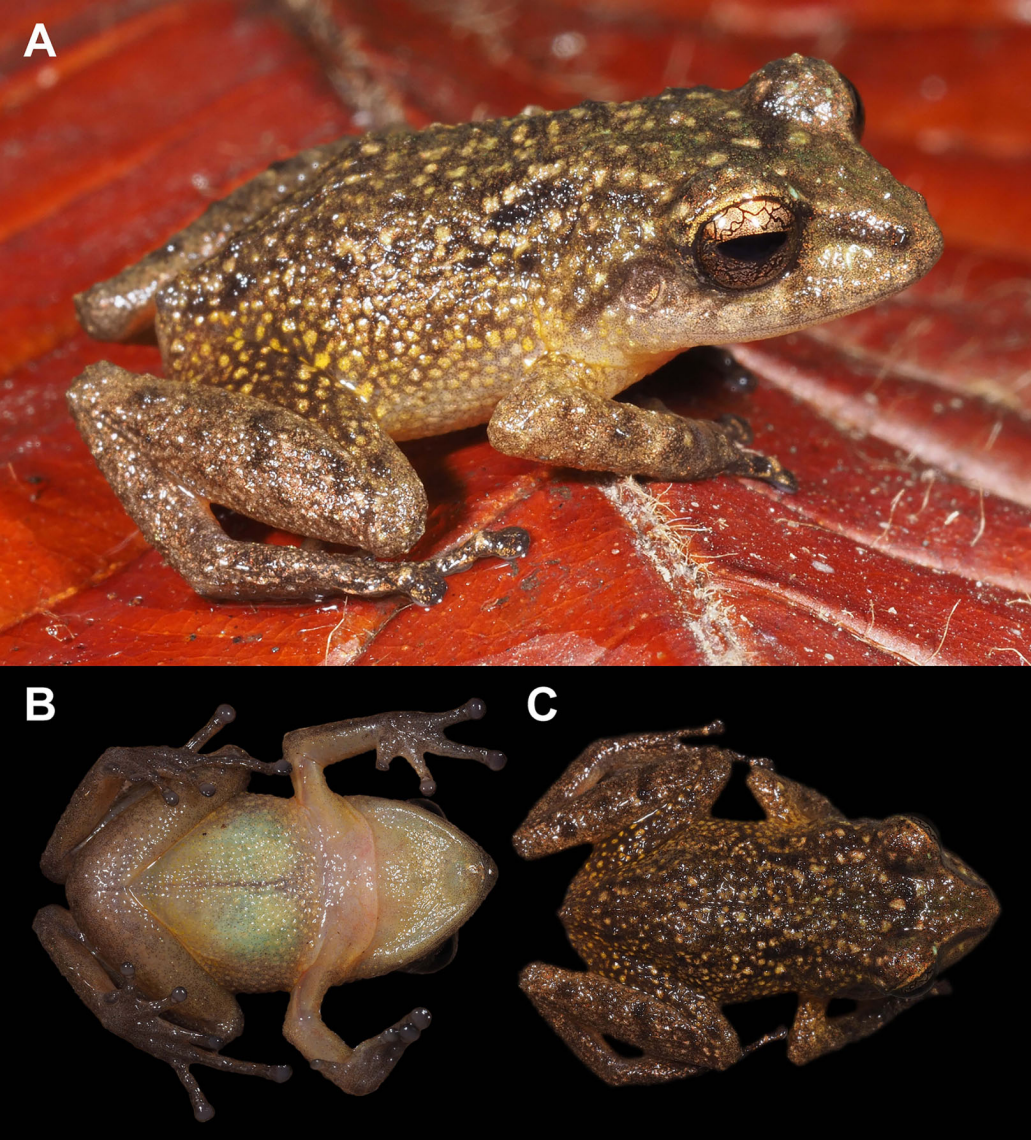
Holotype of *Pristimantis verrucosus* sp. nov. (MUTPL 650; adult male; SVL 20.2 mm) in life, from Reserva Biológica Cerro Plateado, Zamora Chinchipe Province. (A) dorsolateral view; (B) ventral view; (C) dorsal view.

**Figure 3:**
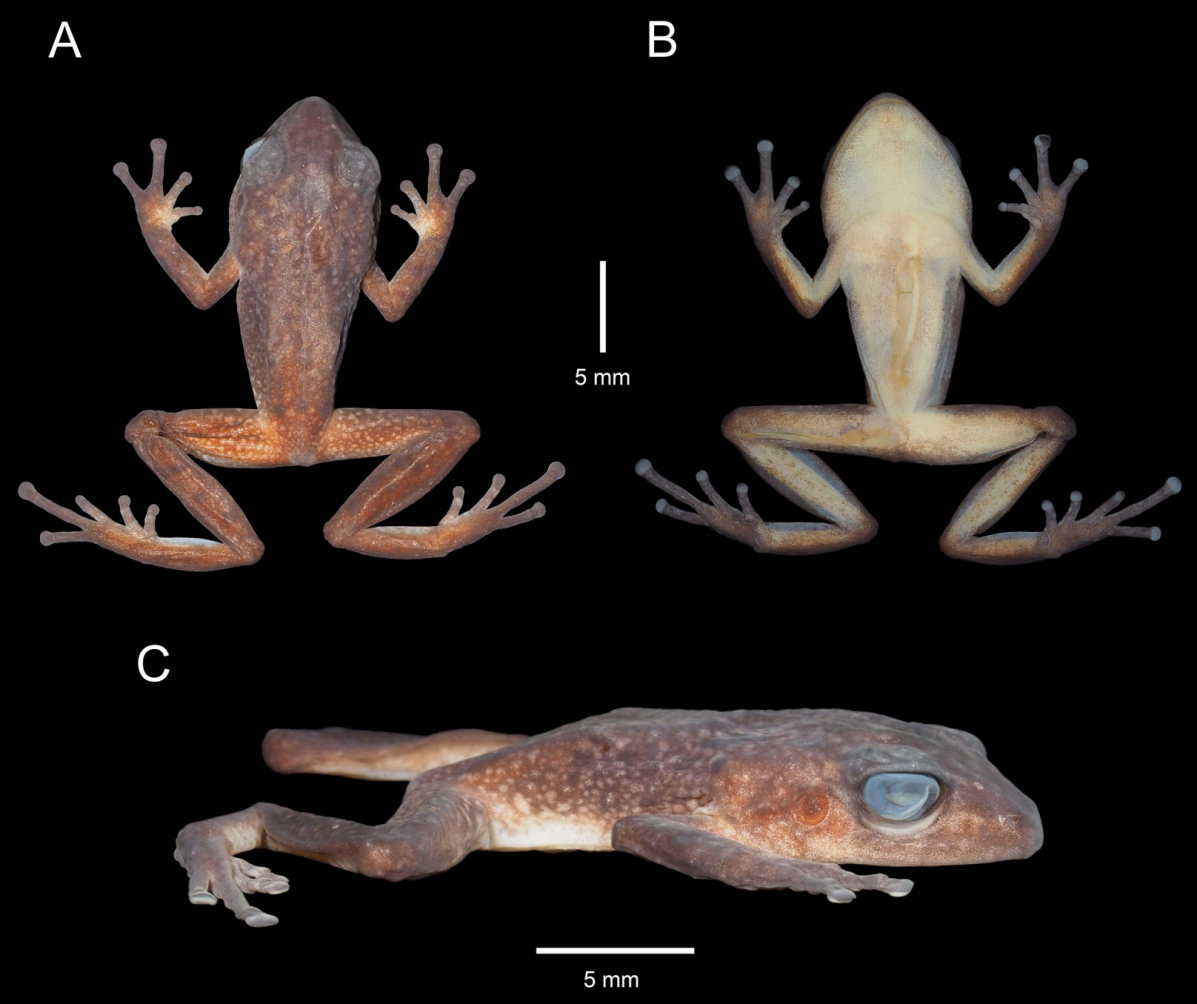
Holotype of *Pristimantis verrucosus* sp. nov. (MUTPL 650; adult male; SVL 20.2 mm) in preservative, from Reserva Biológica Cerro Plateado, Zamora Chinchipe Province. (A) dorsal view; (B) ventral view; (C) lateral view.

**Figure 4:**
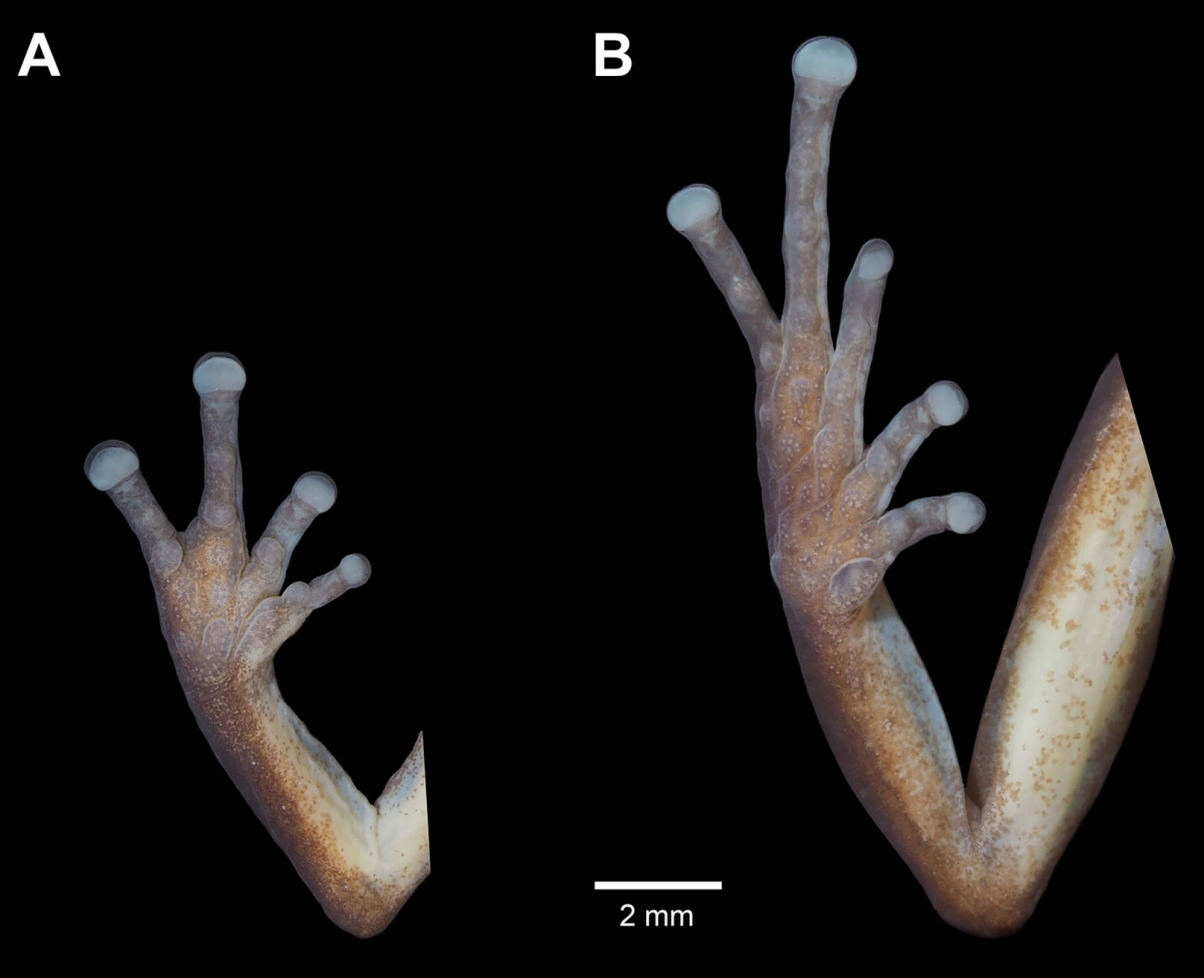
Details of the palmar surface of the hand (A) and plantar surface of the foot (B) of male holotype *Pristimantis verrucosus* sp. nov. (MUTPL 650; SVL 20.2 mm) in preservative.

**Figure 5:**
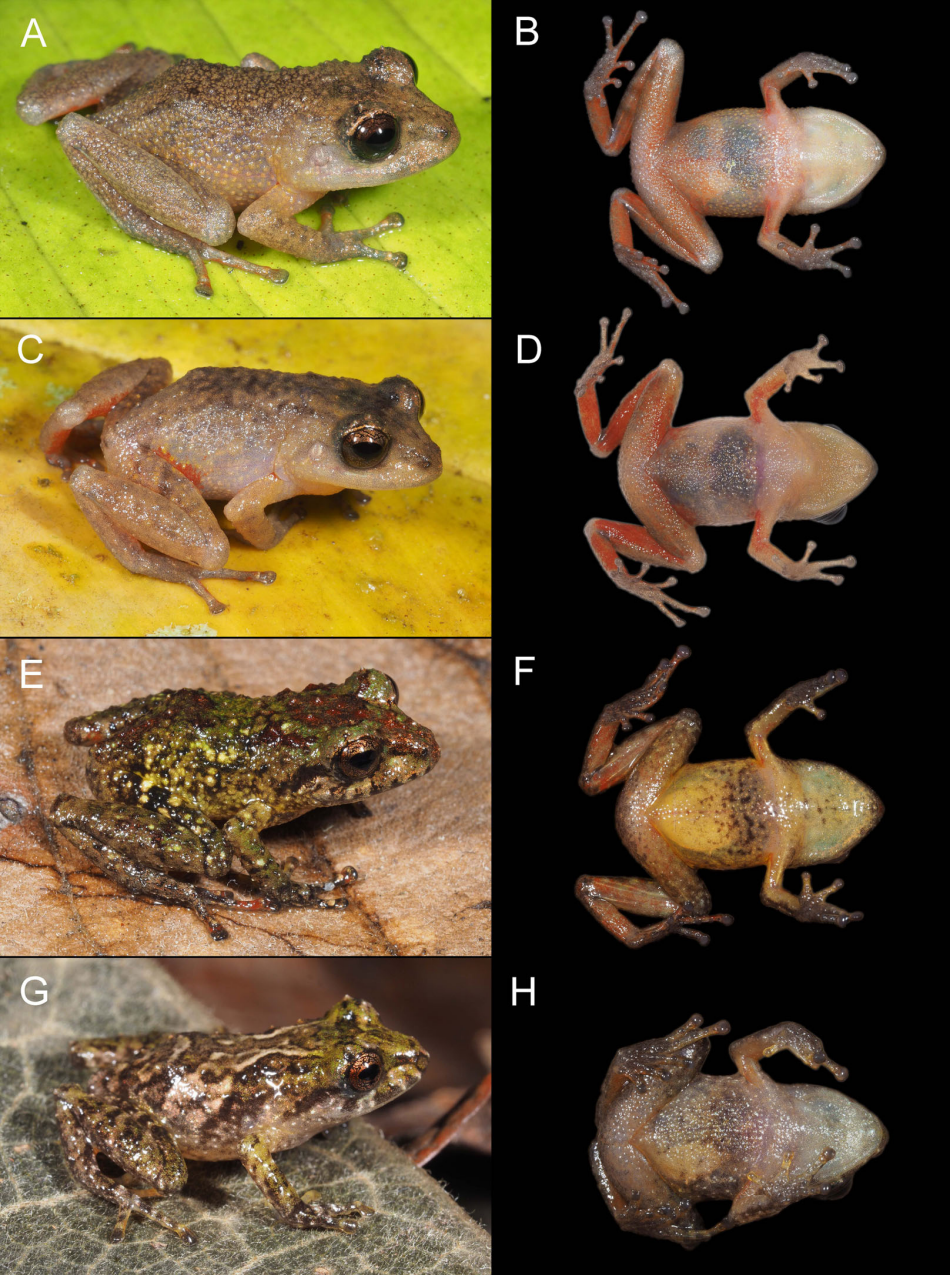
Color variation in life for individuals of *Pristimantis verrucosus* sp. nov., in dorsolateral and ventral views; all individuals are from Reserva Biológica Cerro Plateado, Zamora Chinchipe Province. (A and B) MUTPL 2014, SVL 24.9 mm, adult female paratype; (C and D) MUTPL 2017, SVL 18.8 mm, adult male paratype; (E and F) MUTPL 651, SVL 19.1 mm, subadult female paratype; (G and H) MUTPL 647, SVL 15.3 mm, juvenile paratype.

***Pristimantis verrucosus* sp. nov.**

(Figs. 2, 3, 4 and 5)

ZooBank registration: urn:lsid:zoobank.org:act:59E22EC4-1087-4CB0-B79F-92028D16D618

**Common English name**. Warty Rain Frog

**Common Spanish name.** Cutín verrugoso

**Etymology**. The specific epithet *verrucosus* is derived from Latin, meaning “warty” or “covered with warts”. It refers to the highly tuberculate texture of the dorsum and flanks, which is one of the most distinctive morphological traits of this species. The name is used as an adjective in the masculine form.

**Holotype**. MUTPL 650 (field no. SC 404; Figs. 2, 3 and 4), an adult male from Ecuador, Zamora Chinchipe Province, Reserva Biológica Cerro Plateado (4.6550°S, 78.8733°W; datum WGS84), 2,388 m a.s.l., collected by Paul Székely, Diana Székely, and Santiago Hualpa-Vega on 29 March 2019.

**Paratypes**. (5: 1 female, 2 males, 1 subadult and 1 juvenile, all from Zamora Chinchipe Province, Reserva Biológica Cerro Plateado). MUTPL 651 (field no. SC 405), a subadult female collected with the holotype; MUTPL 647 (field no. SC 401), a juvenile (4.6476°S, 78.8778°W), 2,535 m a.s.l., collected by Paul Székely, Diana Székely, and Santiago Hualpa-Vega on 27 March 2019; MUTPL 2014 (field no. SC 4137), an adult female, and MUTPL 2015 (field no. SC 4143) and MUTPL 2017 (field no. SC 4145) two adult males (4.6467°S, 78.8772°W), 2,516 m a.s.l., collected by Paul Székely, Diana Székely, and Daniel Hualpa-Vega on 25 September 2025.

**Definition**. We assign this species to *Pristimantis* based on phylogenetic evidence (Fig. 1) and on the general morphological similarity to other members of the genus. *Pristimantis verrucosus* is a small-sized species (among the *Huicundomantis* subgenus; *sensu* Székely et al., 2026) characterized by the following combination of characters: (1) skin on dorsum and flanks coarsely tuberculate; skin on venter areolate; discoidal fold present; dorsolateral folds absent; low middorsal fold present; (2) tympanic annulus evident and tympanic membrane differentiated, its length about 44% of the length of eye; supratympanic fold present, completely concealing the upper and posterior edge of tympanum; (3) snout acuminate in dorsal view, rounded in profile; canthus rostralis slightly concave in dorsal view, rounded in profile; (4) upper eyelid bearing one or two larger tubercles (trait more evident in life), about 87% IOD in females and 89% IOD in males; low cranial crests present; (5) dentigerous processes of vomers oblique, ovoid to triangular, separated medially by distance equal to or smaller than the width of processes; each process bearing three to five teeth; (6) males with unpigmented subgular vocal sac, vocal slits and white nuptial pads; (7) fingers relatively short; Finger I shorter than Finger II; discs on fingers slightly expanded, rounded; circumferential grooves present; (8) fingers with lateral fringes; subarticular tubercles prominent; hyperdistal subarticular tubercles present; supernumerary palmar tubercles prominent; palmar tubercle partially divided into a larger (inner) and a smaller (outer) tubercles; thenar tubercle ovoid, larger than the inner palmar tubercle; (9) ulnar tubercles present; (10) heel with two or three slightly larger tubercles (trait more evident in life); outer edge of tarsus with a row of low tubercles (trait more evident in life); inner tarsal fold absent; (11) inner metatarsal tubercle ovoid, about 3 to 5× the size of the elliptical outer metatarsal tubercle; subarticular tubercles prominent; hyperdistal subarticular tubercles present; supernumerary plantar tubercles prominent; (12) toes bearing lateral fringes; webbing basal; Toe V longer than Toe III; discs on toes slightly expanded, rounded, about same size as those on fingers; circumferential grooves present; (13) in life, dorsum and flanks yellowish brown, dark brown to reddish brown or green, with tubercles that vary in coloration, ranging from yellow to brown; venter yellowish white to pinkish white; in some individuals, the ventral surfaces of the axilla, thighs, groin, and concealed limb areas have a reddish orange coloration; iris bronze, with the lower half exhibiting a reddish hue due to a broad median reddish streak, with thin black reticulations; (14) SVL 24.9 mm in one adult female and 18.8–20.2 mm in adult males (19.7 ± 0.78, *n* = 3).

**Comparison with similar species**. *Pristimantis verrucosus* is easily distinguished from most of the Ecuadorian and northern Peruvian species and congeners from the *Huicundomantis* subgenus (and especially from the species of the *P. cryptomelas* group) due to its coarsely tuberculated dorsum and flanks. Only a few Ecuadorian species have dorsum and flanks uniformly covered with tubercles as prominently as *P. verrucosus*.

Among distantly related species, *P. rubicundus* (Jiménez de la Espada, 1875) has a coarsely tuberculate dorsum, but differs markedly in general habitus, with larger and pointier snout, a more prominent tympanum, and larger, more irregular tubercles, including conspicuous tubercles on the heels. Additionally, *P. rubicundus* exhibits a vague outline of W-shaped occipital ridges (*vs*. absent in *P. verrucosus*) and males lack vocal sac and slits (*vs*. males with vocal sac and slits in *P. verrucosus*). Another species that bears some resemblance to *P. verrucosus* is *P. paquishae* Brito, Batallas & Velalcázar, 2014. However, *P. paquishae* can be distinguished by the presence of dorsolateral folds (*vs*. absent in *P. verrucosus*), the fact that males lack vocal slits and nuptial pads (*vs*. males with vocal slits and nuptial pads in *P. verrucosus*), and the iris is grayish with black reticulations (*vs*. iris bronze in *P. verrucosus*); also, the advertisement calls of the two species are markedly different.

Some species that are genetically closer within the subgenus *Huicundomantis* possess tuberculate (or pustulated) dorsum or flanks and may appear superficially similar to *P. verrucosus*, but they can be readily distinguished by the following traits (characters of *P. verrucosus* in parenthesis): *P. balionotus* (Lynch, 1979) has a dorsum covered with black flecks, lacks cranial crests and Toe V is slightly longer than Toe III (without black flecks, with low cranial crests, and Toe V is longer than Toe III); *P. gloria* Páez & Ron, 2019 lacks cranial crests and has broad, black reticulations in the iris (with low cranial crests and thin, black reticulations in the iris); *P. percultus* has larger pustules on the flanks, males lack vocal sac and slits, and Toe V is much longer than Toe III (dorsum and flanks uniformly covered with tubercles, males with vocal sac and slits, and Toe V is longer than Toe III); *P. philipi* lacks tympanum annulus and membrane, and lacks cranial crests (with tympanum annulus and membrane, and with low cranial crests); *P. teslai* Páez & Ron, 2019 lacks a middorsal fold and cranial crests, usually Toe V is much longer than Toe III, and the iris has a copper coloration (with middorsal fold and low cranial crests, Toe V is longer than Toe III, and iris is bronze); *P. verrucolatus* Páez & Ron, 2019 has larger tubercles on the flanks, lacks cranial crests, usually Toe V is much longer than Toe III, and the iris has a copper coloration (dorsum and flanks uniformly covered with tubercles, with low cranial crests, Toe V is longer than Toe III, and iris is bronze). Finally, the highly distinctive advertisement call of *P. verrucosus* clearly sets it apart from all its Ecuadorian and northern Peruvian congeners (see call description below).

**Description of the holotype**. Adult male (MUTPL 650; Figs. 2, 3 and 4), head wider than body, wider than long, HL 84% of HW, HW 41% of SVL, HL 35% of SVL; snout long (EN 11% of SVL; EN 31% of HL), acuminate in dorsal view and rounded in profile; canthus rostralis slightly concave in dorsal view, rounded in profile; loreal region slightly concave; ED larger than EN; nostrils slightly protuberant, oriented posteriorly; lips not flared; low cranial crests are visible; upper eyelid bearing one larger tubercle (trait more visible in life; Fig. 2A); EW 88% of IOD; tympanic annulus evident and tympanic membrane differentiated; supratympanic fold present, completely concealing the upper and posterior margin of the tympanum (Fig. 2A); TD 44% of ED; two postrictal tubercles, one larger than the other, both rounded and low; choanae large, round, not concealed by palatal shelf of maxillary arch; dentigerous processes of vomers prominent, larger than the choanae, oblique, situated posterior and median to choanae, triangular in outline, separated medially by distance smaller than the width of processes, each process bearing four teeth; tongue longer than wider, not notched posteriorly, posterior half not adherent to floor of mouth; large vocal slits near the margin of jaw; subgular vocal sac unpigmented/translucent (Fig. 2B).

Skin on dorsum and flanks coarsely tuberculate; middorsal fold present; dorsolateral folds absent; skin on belly areolate; thoracic and discoidal folds present; cloacal region without ornamentation or evident tubercles.

Ulnar tubercles present; outer palmar tubercle prominent, partially divided into a larger (inner) and a smaller (outer) tubercles; thenar tubercle ovoid, larger than the inner palmar tubercle; subarticular tubercles prominent, round and rounded in section; hyperdistal subarticular tubercles present on all fingers; supernumerary palmar tubercles large, rounded, slightly smaller than subarticular tubercles; inconspicuous, white nuptial pads present at the base of Finger I; fingers bearing lateral fringes; relative length of fingers I < II < IV < III; discs on fingers slightly expanded, rounded; all fingers bearing pads well defined by circumferential grooves (Fig. 4A).

Hindlimbs long (Fig. 3); THL 50% of SVL; TL 52% of SVL; FL 44% of SVL; heel with two slightly larger tubercles (trait more evident in life); outer edge of tarsus with a row of low tubercles (trait more evident in life); inner edge of tarsus without a fold; inner metatarsal tubercle ovoid, about 5× elliptical outer metatarsal tubercle; subarticular tubercles prominent, round and rounded in section; hyperdistal subarticular tubercles present on all toes; supernumerary plantar tubercles large, rounded, slightly smaller than subarticular tubercles; toes bearing lateral fringes; webbing basal; discs on toes slightly expanded, rounded, slightly smaller than those on fingers; toes with ventral pads well defined by circumferential grooves (Fig. 4B); relative length of toes I < II < III < V < IV; Toe V longer than Toe III (tip of Toe III extends beyond proximal edge of penultimate subarticular tubercle on Toe IV, tip of Toe V barely extends beyond proximal edge and doesn’t reach the distal edge of distal subarticular tubercle on Toe IV).

**Coloration of holotype**. In life (Fig. 2): dorsum brown to dark brown, with yellow to light yellow tubercles; the tubercles on the lower flanks, dorsal surfaces of thighs and groin are noticeably more yellowish than those on the dorsum; the dorsal surfaces of hindlimbs and arms light brown; head with faint, light brown, canthal and supratympanic stripes; throat, venter, ventral surfaces of hindlimbs and arms yellowish white; chest pinkish white; iris bronze, with the lower half exhibiting a reddish hue due to a broad median reddish streak, with thin black reticulations.

In preservative (Fig. 3): dorsum reddish brown; dorsal surfaces of arms and of hindlimbs light brown to reddish brown with yellowish spots; flanks brown with yellowish spots; throat, venter, ventral surfaces of hindlimbs and arms yellowish white.

**Measurements of holotype (in mm)**. SVL 20.2; HW 8.3; HL 7.0; IOD 2.5; IND 1.8; EW 2.2; ED 2.7; EN 2.2; TD 1.2; THL 10.0; TL 10.5; FL 8.9; FLL 5.3; HAL 5.5.

**Body mass of holotype**. 0.82 g.

**Variation**. Morphometric variation is shown in Table 1. Coloration does not differ between sexes. The female MUTPL 2014 (Figs. 5A and 5B) and male MUTPL 2017 (Figs. 5C and 5D) had the ventral surfaces of the axilla, thighs, groin, and concealed limb areas reddish orange. None of the paratypes had tubercles on the dorsum or flanks as yellow as those of the holotype. Subadult MUTPL 651 (Figs. 5E and 5F) exhibited more colorful dorsum and flanks, displaying a camouflage pattern composed of green, reddish brown, yellow, and light brown patches. The venter of this individual was yellowish orange, contrasting with the yellowish or pinkish white venters observed in the other specimens. Juvenile MUTPL 647 (Fig. 5G) exhibited a less tuberculated dorsum and a dorsal pattern closely resembling that of paratype MUTPL 2015, characterized by cream-colored reticulum or lines.

**Table 1:** Body measurements of adults of the three new *Pristimantis* species. Body mass (in grams), measurements (in mm) and morphological proportions (in percentages). Values are given as mean ± SD (range). Female body mass includes eggs.

Character	*Pristimantis verrucosus* sp. nov.		*Pristimantis plateado* sp. nov.		*Pristimantis melanops* sp. nov.	
	Females (*n* = 1)	Males (*n* = 3)	Females (*n* = 2)	Males (*n* = 2)	Females (*n* = 3)	Males (*n* = 3)
Body mass (BM)	1.14	0.72 ± 0.11 (0.61–0.82)	1.55–2.28	0.64–1.09	1.27 ± 0.24 (1.12–1.54)	0.78 ± 0.07 (0.70–0.83)
Snout-vent length (SVL)	24.9	19.7 ± 0.78 (18.8–20.2)	27.2–29.9	20.2–23.0	24.5 ± 0.67 (23.9–25.2)	20.3 ± 0.72 (19.5–20.8)
Head width (HW)	10.1	8.1 ± 0.35 (7.7–8.3)	10.8–12.8	7.7–9.1	9.2 ± 0.40 (8.8–9.6)	7.8 ± 0.06 (7.8–7.9)
Head length (HL)	9.1	7.0 ± 0.10 (6.9–7.1)	10.2–11.2	7.3–8.5	8.3 ± 0.12 (8.2–8.4)	7.1 ± 0.15 (6.9–7.2)
Interorbital distance (IOD)	3.1	2.4 ± 0.58 (2.4–2.5)	3.5–3.8	2.5–2.8	3.1 ± 0.12 (3.0–3.2)	2.5 ± 0.20 (2.3–2.7)
Internarial distance (IND)	2.1	1.8 ± 0.06 (1.7–1.8)	2.3–2.7	1.7–2.2	2.1 ± 0.10 (2.0–2.2)	1.8 ± 0.10 (1.7–1.9)
Upper eyelid width (EW)	2.7	2.2 ± 0.06 (2.1–2.2)	2.9–3.0	2.2–2.4	2.4 ± 0.21 (2.2–2.6)	2.3 ± 0.10 (2.2–2.4)
Eye diameter (ED)	3.2	2.8 ± 0.10 (2.7–2.9)	3.8–3.9	2.8–3.3	3.1 ± 0.10 (3.0–3.2)	3.0 ± 0.12 (2.9–3.1)
Eye-nostril distance (EN)	2.8	2.1 ± 0.10 (2.0–2.2)	3.1–3.6	2.1–2.4	2.9 ± 0.06 (2.8–2.9)	2.2 ± 0.06 (2.2–2.3)
Tympanum diameter (TD)	1.5	1.2 ± 0.10 (1.1–1.3)	1.4–1.8	1.0–1.1	1.7 ± 0.12 (1.6–1.8)	1.1 ± 0.06 (1.1–1.2)
Thigh length (THL)	12.4	9.8 ± 0.21 (9.6–10.0)	14.4–14.9	10.0–12.0	11.6 ± 0.25 (11.3–11.8)	9.5 ± 0.26 (9.3–9.8)
Tibia length (TL)	13.1	10.1 ± 0.36 (9.8–10.5)	15.2–15.5	10.7–13.1	12.5 ± 0.26 (12.2–12.7)	10.3 ± 0.40 (9.9–10.7)
Foot length (FL)	10.7	8.8 ± 0.36 (8.4–9.1)	13.1–13.8	9.4–10.5	10.1 ± 0.26 (9.8–10.3)	8.23 ± 0.38 (7.8–8.5)
Forearm length (FLL)	6.4	5.1 ± 0.15 (5.0–5.3)	5.2–6.0	5.2–6.0	5.5 ± 0.36 (5.2–5.9)	4.6 ± 0.10 (4.5–4.7)
Hand length (HAL)	6.8	5.5 ± 0.30 (5.2–5.8)	8.0–8.7	5.6–6.9	6.2 ± 0.15 (6.1–6.4)	5.1 ± 0.21 (4.9–5.3)
HW/SVL	40.6	41.0–41.3	39.7–42.8	38.1–39.6	36.2–38.1	37.7–40.0
HL/SVL	36.5	34.7–36.7	37.5	36.1–37.0	33.3–34.3	34.1–35.4
EN/SVL	11.2	10.4–10.9	11.4–12.0	10.4	11.1–12.1	10.6–11.3
HL/HW	90.1	84.3–89.6	87.5–94.4	93.4–94.8	87.5–93.2	88.5–92.3
EN/HL	30.8	29.0–31.4	30.4–32.1	28.2–28.8	33.3–35.4	31.0–31.9
ED/HL	35.2	38.6–42.0	34.8–37.3	38.4–38.8	36.6–38.1	40.8–43.1
EW/IOD	87.1	87.5–91.7	78.9–82.9	85.7–88.0	68.8–81.3	88.9–95.7
EN/ED	87.5	60.0–81.5	81.6–92.3	72.7–75.0	87.5–96.7	74.2–75.9
TD/ED	46.9	37.9–46.4	36.8–46.2	33.3–35.7	53.3–58.1	35.5–41.4
THL/SVL	49.8	49.3–51.1	49.8–52.9	49.5–52.2	46.0–49.4	44.9–48.2
TL/SVL	52.6	49.8–52.1	51.8–55.9	53.0–57.0	48.4–52.7	50.0–51.7
FL/SVL	43.0	44.1–45.3	46.2–48.2	45.7–46.5	40.3–42.7	40.0–40.9
FLL/SVL	25.7	25.4–26.6	25.4–26.1	23.8–25.7	20.6–24.3	22.2–23.1
HAL/SVL	27.3	27.2–28.9	29.1–29.4	27.7–30.0	24.2–26.8	25.1–25.5

**Vocalizations**. For the description of the *P. verrucosus* advertisement call, we analyzed four call samples from four different males, all recorded in Reserva Biológica Cerro Plateado, Zamora Chinchipe Province, in September 2025. Additionally, we describe one distress call sample, recorded in the laboratory. Detailed information for each of the recordings is provided in File S2.

*Pristimantis verrucosus* has a highly distinctive and peculiar advertisement call, characterized by a series of screeching sounds (notes) repeated over time. The vocalization is unique among known congeners from Southern Ecuador and northern Peru, and bears resemblance only to an undescribed species, sister of *P. nangaritza*, from Reserva Tapichalaca (P. Székely, 2016, personal observation). Notably, this is the first advertisement call described for a species within the *P. cryptomelas* group, and we hypothesize that up to half of the species in this group may be mute, as most lack vocal slits (Coloma & Duellman, 2025).

**Figure 6:**
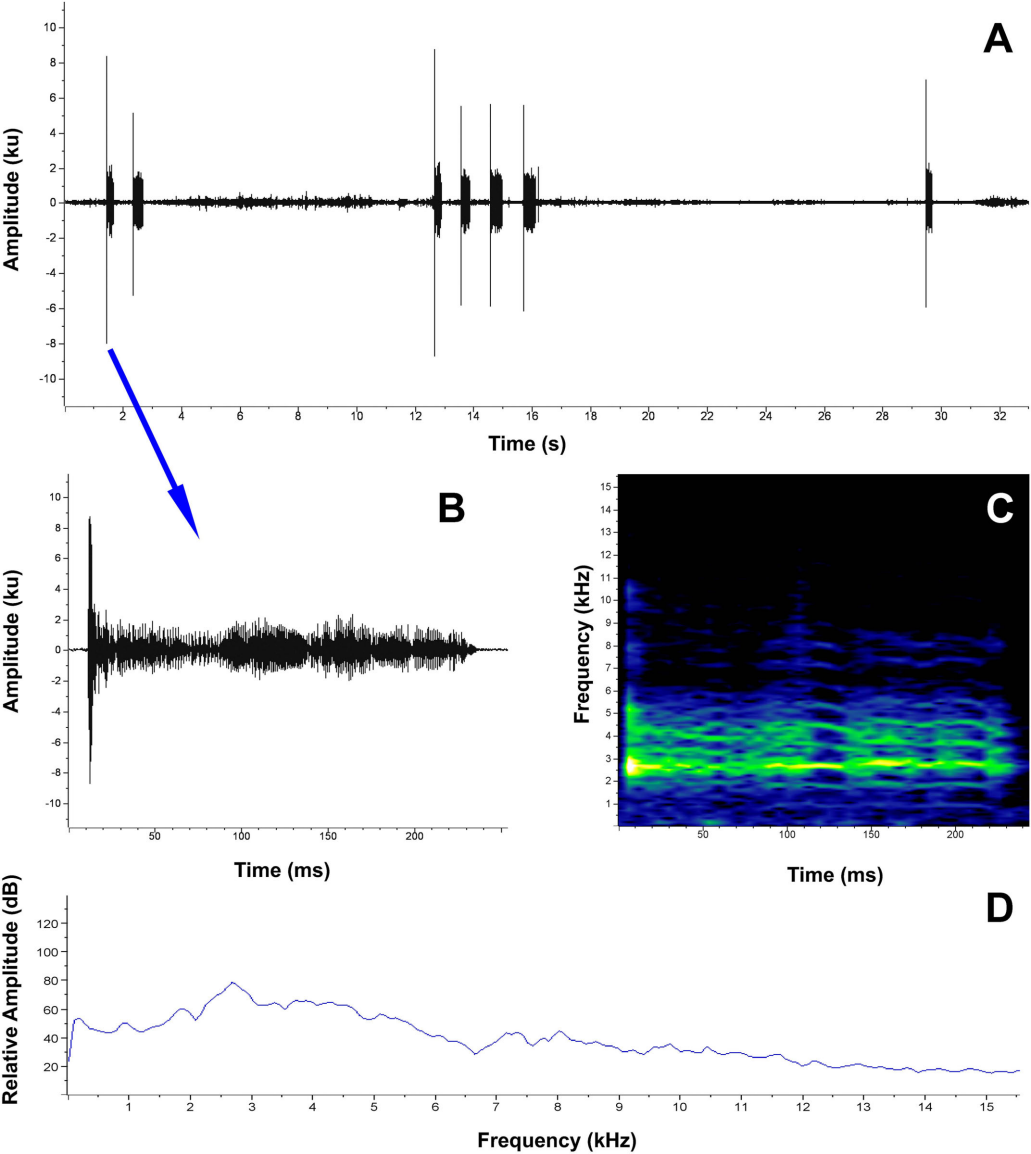
Advertisement call of *Pristimantis verrucosus* sp. nov. (paratype MUTPL 2017, FUTPL-A-1195). (A) Oscillogram of three consecutive calls, the first with two notes, the second with four and the third with one note; (B) oscillogram of a note; (C) spectrogram of a note; (D) power spectrum of a note.

The calls are typically composed of two notes, however, in some instances, the call may contain up to four distinct notes (Fig. 6). The double-noted calls are characterized by durations of 0.980–1.384 s (1.246 ± 0.18, *n* = 4), while the only recorded four-noted call (FUTPL-A-1195) lasted 3.456 s. The inter-call interval has a duration of 7.242–11.262 s (9.715 ± 1.74, *n* = 4), and the call rate is of 3.81–5.42 calls/min (4.67 ± 0.75, *n* = 4). The notes are pulsatile sounds (as no distinct pulses can be detected), relatively long, characterized by a duration of 0.135–0.237 s (0.164 ± 0.05, *n* = 4), an inter-note interval of 0.701–1.034 s (0.880 ± 0.16, *n* = 4) and a note rate of 0.86–1.21 notes/s (0.98 ± 0.16, *n* = 4). The notes within each call are structurally and temporally very similar, although the final notes in multi-note calls tend to be longer. Thus, in the four-note call (Fig. 6A), the first note had a duration of 0.225 s, the second 0.302 s, the third 0.410 s, and the fourth 0.408 s. The mean dominant frequency of the calls was 2,473.2–2,670.1 Hz (2,551.9 ± 93.59, *n* = 4), with a mean 90% bandwidth that ranged from 2,214.8–2,411.7 Hz (2,335.6 ± 89.55, *n* = 4) to 2,842.4–4,108.5 Hz (3,290.6 ± 561.14, *n* = 4). The fundamental frequency is not clearly recognizable, and the harmonics appear scrambled and difficult to distinguish, which complicates the spectral characterization of the call (Fig. 6C).

During the photographic documentation of the collected specimens in the laboratory, three individuals (two in 2019 and one in 2025) emitted a series of high-pitched, whining vocalizations, which we considered distress calls. These calls are typically produced when frogs are seized by predators or while attempting to escape from them (Wells, 2007; Toledo et al., 2015). Four distress calls (FUTPL-A-1194) were recorded in 2025 after one specimen (paratype MUTPL 2015) was unexpectedly stimulated by contact with a tweezer on one of its toes, which appeared to startle the animal and triggered the vocalizations. The animal emitted the calls with its mouth closed and without displaying any defensive posture or behavior.

**Figure 7:**
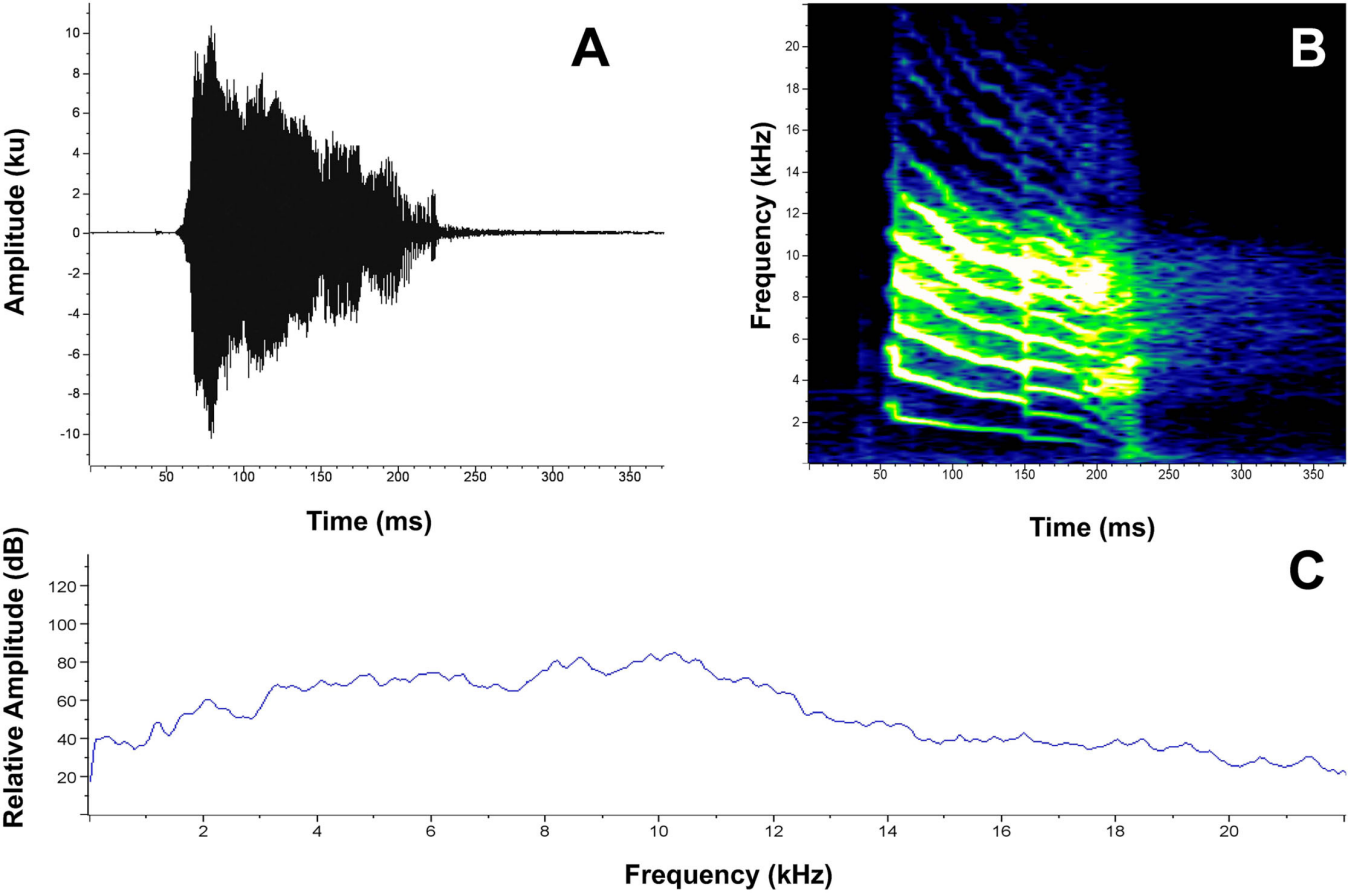
Distress call of *Pristimantis verrucosus* sp. nov. (paratype MUTPL 2015, FUTPL-A-1194). (A) Oscillogram of a note; (B) spectrogram of a note; (C) power spectrum of a note.

The distress call of *P. verrucosus* (Fig. 7) is characterized by a short, high-pitched note, with decreasing frequency modulation, a duration of 0.186–0.197 s (0.191 ± 0.005, *n* = 4), a dominant frequency of 8,010.4–10,249.8 Hz (9,259.3 ± 936.93, *n* = 4), and a mean 90% bandwidth that ranged from 4,134.4–5,770.9 Hz (4,823.5 ± 823.14, *n* = 4) to 8,699.4–10,766.6 Hz (9,926.8 ± 890.6, *n* = 4). Between 11 and 13 harmonics are visible on the spectrogram, although some may represent sidebands resulting from modulation (Fig. 7).

**Figure 8:**
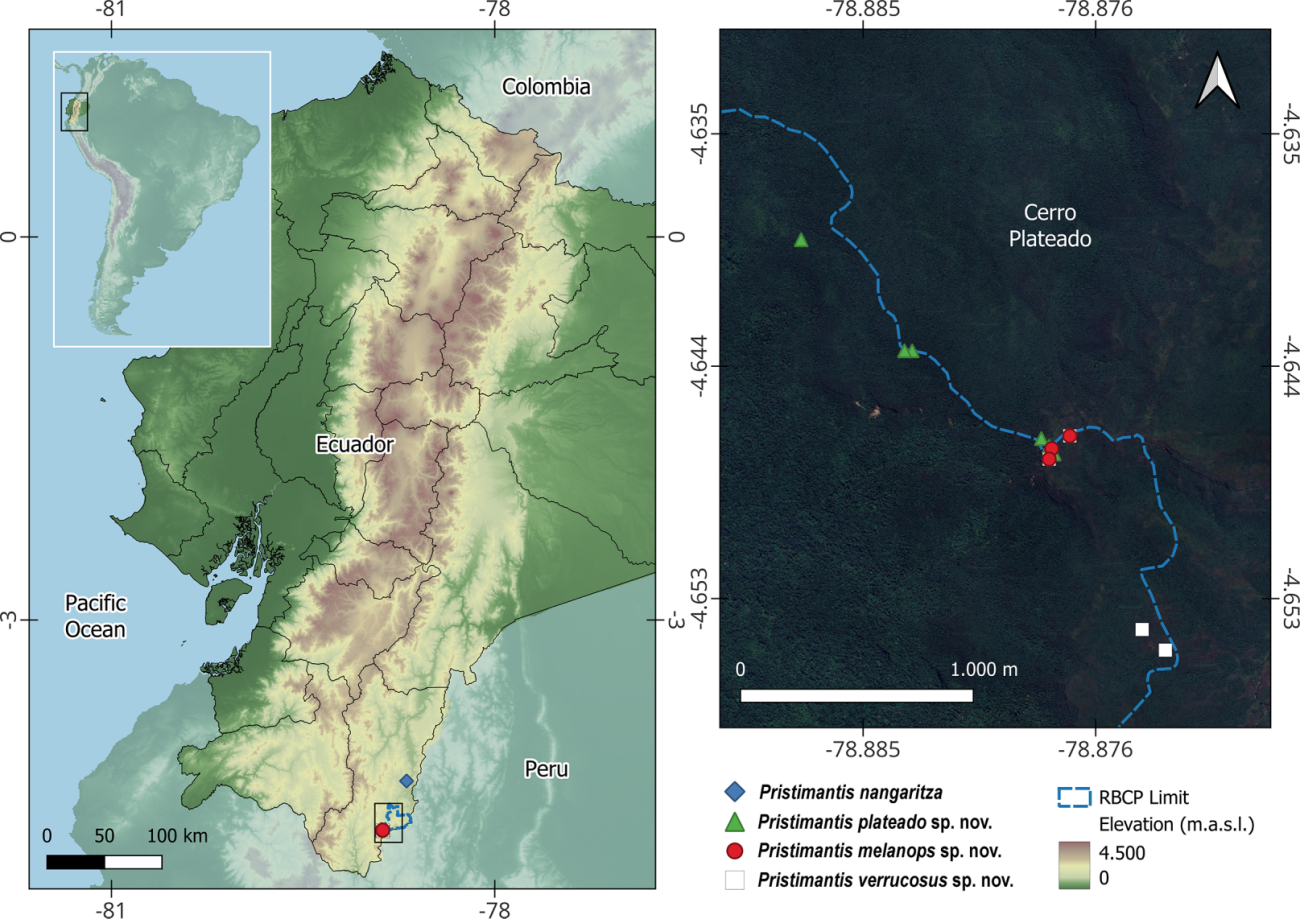
Distribution of the three new *Pristimantis* species and *P. nangaritza*. Records are based on specimens deposited at the Museo de Zoología, Universidad Técnica Particular de Loja (MUTPL), Museo de Zoología, Pontificia Universidad Católica del Ecuador (QCAZ), and authors’ field data. The map on the right shows a zoomed-in view of the area marked by the square in the general map on the left. Satellite image courtesy of Google Earth (https://mt1.google.com/vt/lyrs=s&x={x}&y={y}&z={z}).

**Distribution**. *Pristimantis verrucosus* is currently known only from a single locality, situated within and immediately adjacent to the Reserva Biológica Cerro Plateado (Fig. 8). However, given the proximity of the reserve to the Peruvian border, it is plausible that *P. verrucosus* also occurs in adjacent areas of northern Peru. Specimens were encountered at an altitudinal range between 2,388 and 2,535 m in “Montane evergreen forest on sandstone tablelands of the Cordillera del Cóndor” and “Montane evergreen shrubland and grassland of the Cordillera del Cóndor” ecosystems.

**Natural history.** We consider this species a bromeliad specialist (*sensu* Székely et al., 2026), as all individuals were observed either hidden inside terrestrial bromeliads or calling from their leaves. All animals were encountered during the night; calling males were heard in September. Sympatric frog species include *P. plateado* sp. nov., *P. melanops* sp. nov., an undescribed *Pristimantis* species and an undescribed species of salamander of the genus *Bolitoglossa*.

**Conservation status**. *Pristimantis verrucosus* is known from a single locality, within and immediately adjacent to a protected area, the Reserva Biológica Cerro Plateado, occupying an estimated area of less than 8 km^2^. Although our assessment of the species distribution area may be underestimated due to incomplete sampling of the reserve, the extent of potentially suitable habitat is very unlikely to exceed 25 km^2^, as these frogs occur in an isolated sandstone plateau ecosystem resembling tepuis. Furthermore, neither of the previous expeditions to nearby tepui-like formations inside RBCP reported these species, reinforcing our assumption of a highly restricted range.

Even though it is a protected area, illegal mining activities have been detected both inside its boundaries and in its immediate surroundings. In the vicinity, less than 3 km from the limit of the reserve and within its buffer zone, the Panguri River has already been severely impacted by uncontrolled mining operations. Thus, based on the available information, we recommend that *P. verrucosus* be categorized as Critically Endangered following the B1ab(i,ii,iii,iv)+2ab(i,ii,iii,iv) IUCN criteria (IUCN, 2001) because: (1) its Extent of Occurrence (EOO) and Area of Occupancy (AOO) are estimated to be less than 8 km^2^; (2) it is known from only one location; and (3) its habitats could be affected in the near future by illegal mining activities.

**Figure 9:**
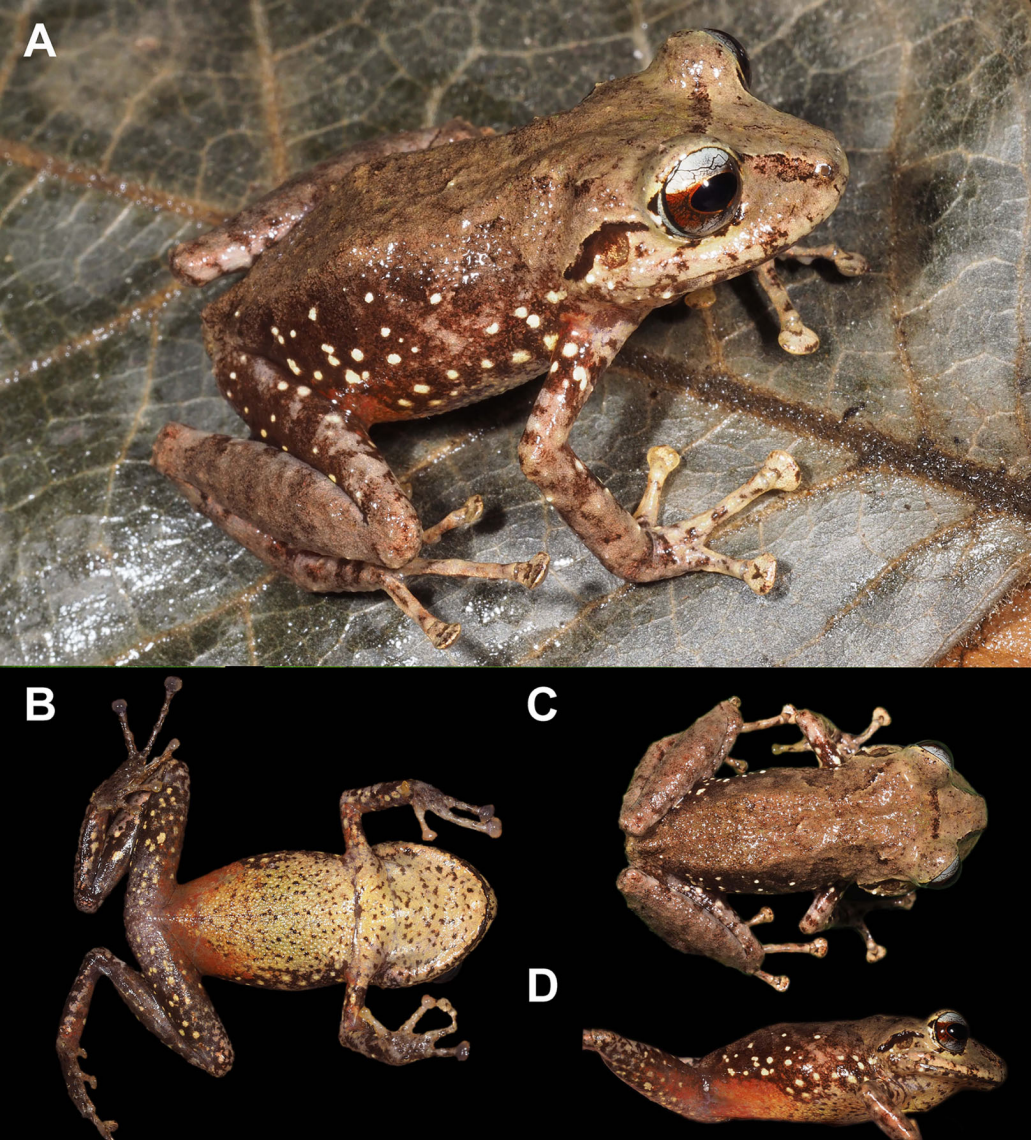
Holotype of *Pristimantis plateado* sp. nov. (MUTPL 648; adult female; SVL 29.9 mm) in life, from Reserva Biológica Cerro Plateado, Zamora Chinchipe Province. (A) dorsolateral view; (B) ventral view; (C) dorsal view: (D) lateral view.

**Figure 10:**
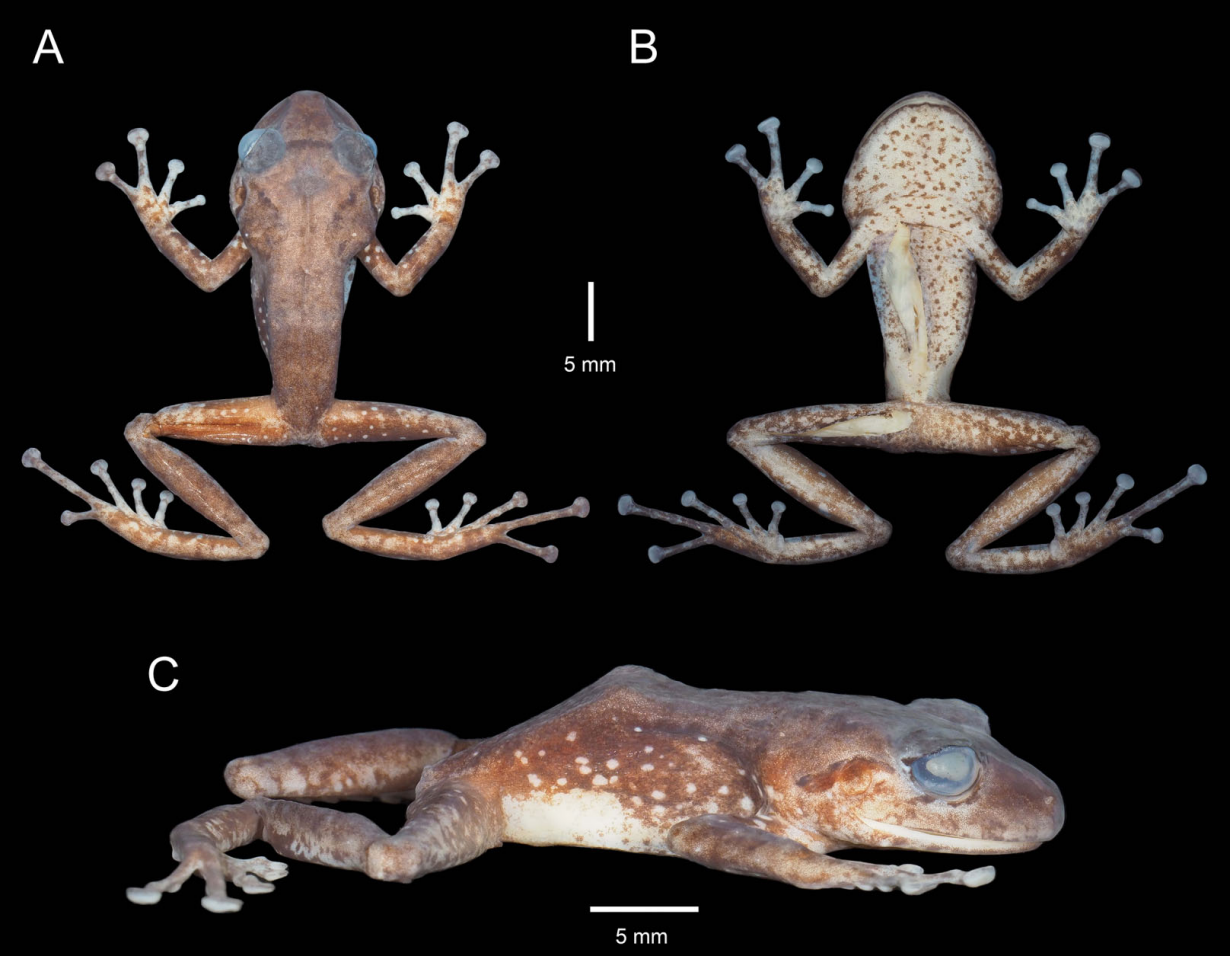
Holotype of *Pristimantis plateado* sp. nov. (MUTPL 648; adult female; SVL 29.9 mm) in preservative, from Reserva Biológica Cerro Plateado, Zamora Chinchipe Province. (A) dorsal view; (B) ventral view; (C) lateral view.

**Figure 11:**
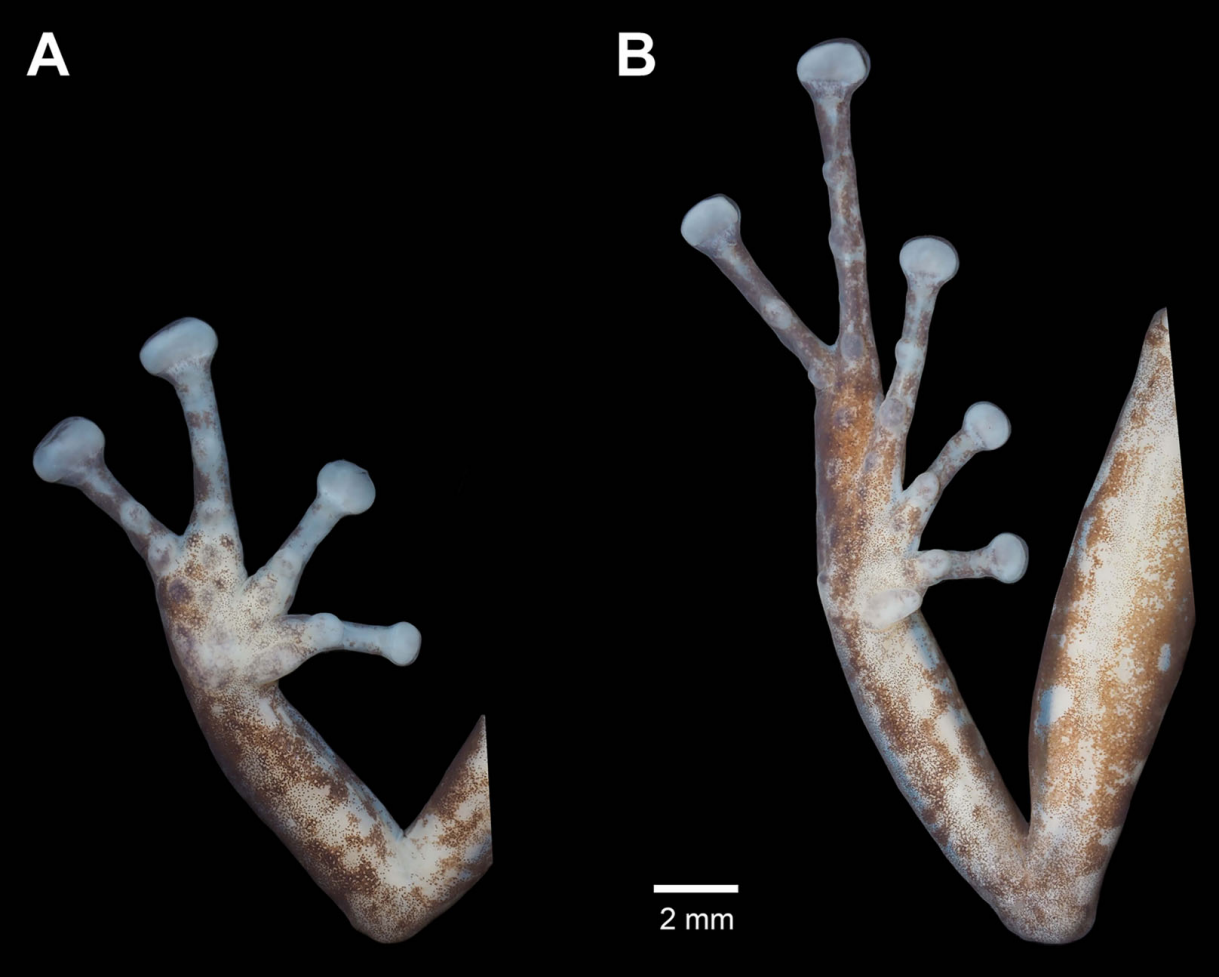
Details of the palmar surface of the hand (A) and plantar surface of the foot (B) of female holotype *Pristimantis plateado* sp. nov. (MUTPL 648; SVL 29.9 mm) in preservative.

**Figure 12:**
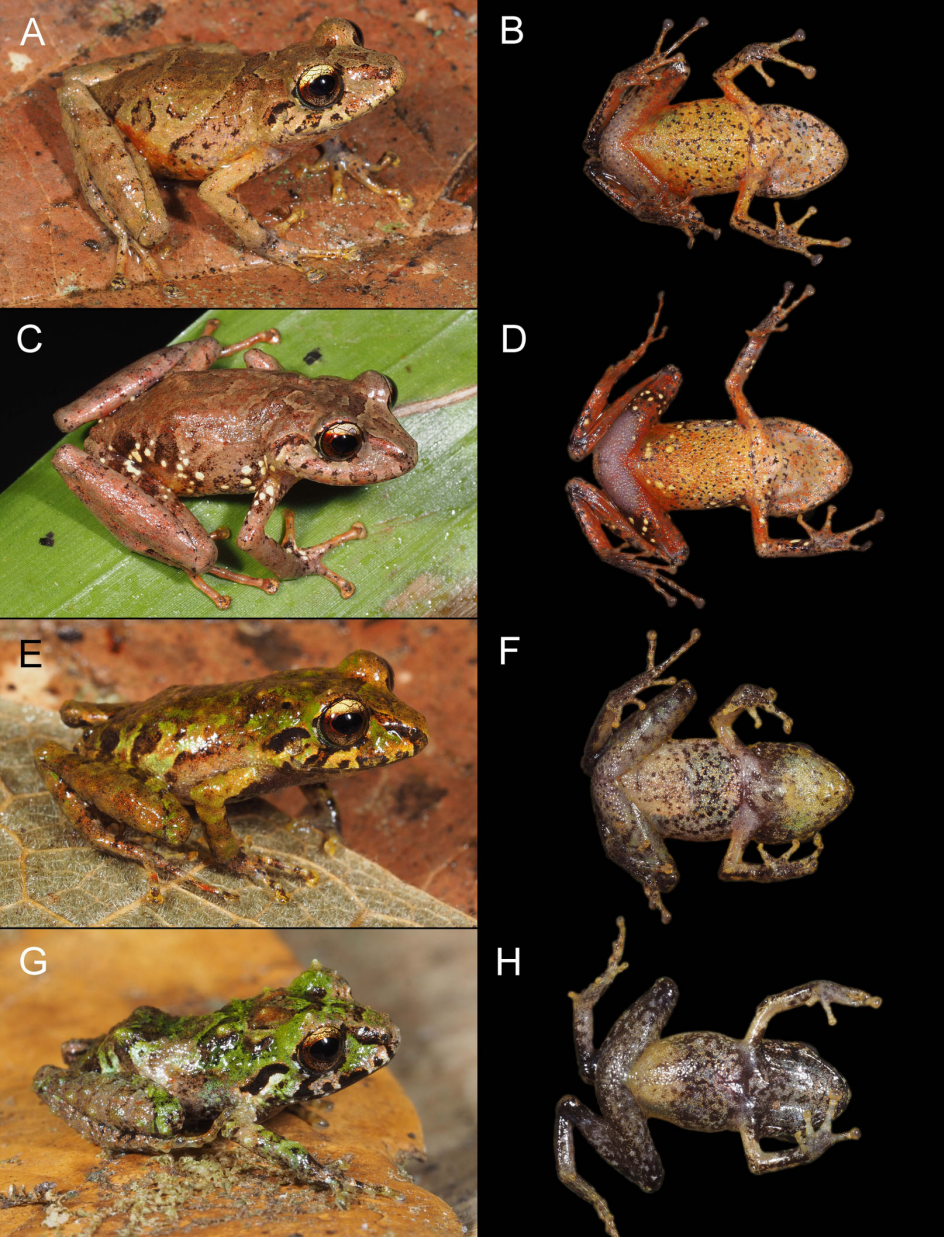
Color variation in life for individuals of *Pristimantis plateado* sp. nov., in dorsolateral and ventral views; all individuals are from Reserva Biológica Cerro Plateado, Zamora Chinchipe Province. (A and B) MUTPL 616, SVL 23.0 mm, adult male paratype; (C and D) MUTPL 610, SVL 27.2 mm, adult female paratype; (E and F) MUTPL 613, SVL 17.7 mm, subadult paratype; (G and H) MUTPL 609, SVL 13.2 mm, juvenile paratype.

***Pristimantis plateado* sp. nov.**

(Figs. 9, 10, 11, and 12)

ZooBank registration: urn:lsid:zoobank.org:act:A15EE2A4-696A-4159-BD8C-A396A840B42A

**Common English name**. Silvery Rain Frog

**Common Spanish name**. Cutín plateado

**Etymology**. The specific epithet *plateado* is a noun in apposition that means “silver” or “silvery” in Spanish. It refers to both the type locality, the Reserva Biológica Cerro Plateado, and a distinctive morphological trait of the species—the silvery iris observed in some individuals. The name of the reserve, Cerro Plateado, can be translated as “Silvery Hill” or “Silvery Mountain”. The reserve includes montane evergreen forests on sandstone plateaus, which may reflect light in a way that gives the landscape a silvery appearance, inspiring its name.

**Holotype**. MUTPL 648 (field no. SC 402; Figs. 9, 10 and 11), an adult female from Ecuador, Zamora Chinchipe Province, Reserva Biológica Cerro Plateado (4.6468°S, 78.8781°W; datum WGS84), 2,512 m a.s.l., collected by Paul Székely, Diana Székely, and Santiago Hualpa-Vega on 27 March 2019.

**Paratypes.** (Five: one female, two males, one subadult and one juvenile, all from Zamora Chinchipe Province, Reserva Biológica Cerro Plateado). MUTPL 609 (field no. SC 364), a juvenile and MUTPL 610 (field no. SC 366), an adult female (4.6474°S, 78.8776°W), 2,534 m a.s.l.; MUTPL 612 (field no. SC 368) a male and MUTPL 613 (field no. SC 369) a subadult (4.6434°S, 78.8831°W), 2,342 m a.s.l.; MUTPL 616 (field no. SC 372), an adult male from (4.6391°S, 78.8874°W), 2,195 m a.s.l., all collected by Santiago Hualpa-Vega and Daniel Hualpa-Vega on 18 January 2019.

**Definition**. We assign this species to *Pristimantis* based on phylogenetic evidence (Fig. 1) and on the general morphological similarity to other members of the genus. *Pristimantis plateado* is a small-sized species (among the *Huicundomantis* subgenus; *sensu* Székely et al., 2026) characterized by the following combination of characters: (1) skin on dorsum and flanks shagreen with scattered tubercles (trait more evident in life); skin on venter areolate; discoidal fold present; dorsolateral folds absent; low middorsal fold present; (2) tympanic annulus evident and tympanic membrane differentiated, its length about 38% of the length of eye; supratympanic fold present, slightly concealing the upper edge of tympanum; (3) snout rounded in dorsal view, rounded in profile; canthus rostralis slightly concave in dorsal view, rounded in profile; (4) upper eyelid bearing one slightly larger tubercle (trait more evident in life), about 81% IOD in females and 87% IOD in males; cranial crests absent; (5) dentigerous processes of vomers oblique, ovoid to triangular, separated medially by distance equal to or smaller than the width of processes; each process bearing three to seven teeth; (6) males lacking vocal sac and slits; (7) Finger I shorter than Finger II; discs on fingers broadly expanded, rounded; circumferential grooves present; (8) fingers with lateral fringes; subarticular tubercles prominent; hyperdistal subarticular tubercles present; supernumerary palmar tubercles prominent; palmar tubercle partially divided into a larger (inner) and a smaller (outer) tubercles; thenar tubercle elliptical, the same size or slightly smaller than the inner palmar tubercle; (9) ulnar tubercles low (trait more evident in life); (10) heel with one slightly larger, rounded tubercle (trait more evident in life); outer edge of tarsus with a row of low tubercles (trait more evident in life); inner tarsal fold absent; (11) inner metatarsal tubercle ovoid, about 4 to 5× the size of the round outer metatarsal tubercle; subarticular tubercles prominent; hyperdistal subarticular tubercles present; supernumerary plantar tubercles prominent; (12) toes bearing lateral fringes; webbing basal; Toe V longer than Toe III; discs on toes broadly expanded, rounded, slightly smaller than those of fingers; circumferential grooves present; (13) in life, dorsum light brown with or without dark brown markings, to brown mottled with green or nearly entirely green; flanks sometimes with white spots; venter yellowish white to orange, with variable black mottling; in some individuals the groin, axilla, and concealed limb surfaces are reddish orange; iris silver to light bronze, with a median, broad red streak and fine black reticulations; (14) SVL 27.2–29.9 mm in adult females (*n* = 2) and SVL 20.2–23.0 mm in adult males (*n* = 2).

**Comparison with similar species**. *Pristimantis plateado* is morphologically similar only to a couple of species within the subgenus *Huicundomantis*, but it can be readily distinguished from all of them, due to the unique combination of morphological traits. It most closely resembles its distant relative from the *P. phoxocephalus* group, the sympatric *P. versicolor* (Lynch, 1979), but it can be primarily distinguished by the following traits (characters of *plateado* in parenthesis): flanks tuberculate (flanks shagreen), longer (EN about 32% of HL), more angular snout, which appears subacuminate in dorsal view and sharply defined with angular canthus rostralis (shorter (EN about 30% of HL), rounded snout in dorsal view and rounded canthus rostralis), a proportionally larger tympanum (TD about 16% of HL; TD about 45% of ED) (smaller tympanum (TD about 14% of HL; TD about 38% of ED)), large tubercle on the heel (small tubercle on the heel), inner tarsal fold present (inner tarsal fold absent), Toe V much longer than Toe III (Toe V longer than Toe III), and the broad reddish median horizontal streak covers the entire lower half of the iris (the red streak is limited to the middle part of the iris).

*Pristimantis plateado* bares only a superficial resemblance to its genetically close, sister species, *P. nangaritza*, from which it is easily distinguished by the presence of )(-shaped postocular folds (*vs*. absent in *P. plateado*), a reddish iris (*vs*. silver or light bronze iris in *P. plateado*; Coloma & Duellman, 2025), and the presence of vocal slits in males (*vs*. absence of vocal slits in *P. plateado* males). Furthermore, *P. nangaritza* is known only from an isolated, tepui-like mountain summit located approximately 47 km north in a straight line (Fig. 8), and occurring at a considerably lower elevation range of 1,809–1,843 m (Coloma & Duellman, 2025).

Finally, *P. plateado* shows some superficial similarity to *P. hampatusami* Yánez-Muñoz, Sánchez-Nivicela & Reyes-Puig, 2016, a species from the western slopes of the Andes, from which it can be readily distinguished by its subacuminate snout in dorsal view (*vs*. rounded in *P. plateado*), the presence of a low W-shaped ridge in the scapular region (*vs*. absent in *P. plateado*), and the presence of a vocal sac and slits in males (*vs*. absence of both in *P. plateado* males).

**Description of the holotype**. Adult female (MUTPL 648; Figs. 9, 10 and 11), head wider than body, wider than long, HL 88% of HW, HW 43% of SVL, HL 38% of SVL; snout relatively short (EN 12% of SVL; EN 32% of HL), rounded in dorsal view and rounded in profile; canthus rostralis slightly concave in dorsal view, rounded in profile; loreal region slightly concave; ED larger than EN; nostrils slightly protuberant, oriented posteriorly; lips not flared; cranial crests absent; upper eyelid bearing one larger tubercle (trait more visible in life; Fig. 9A); EW 79% of IOD; tympanic annulus evident and tympanic membrane differentiated; supratympanic fold present, slightly concealing the upper margin of the tympanum (Fig. 9A); TD 46% of ED; one large, conical postrictal tubercle; choanae large, oval, not concealed by palatal shelf of maxillary arch; dentigerous processes of vomers prominent, larger than the choanae, oblique, situated posterior and median to choanae, ovoid in outline, separated medially by distance smaller than the width of processes, each process bearing five to six teeth; tongue longer than wider, slightly notched posteriorly, posterior half not adherent to mouth floor.

Skin on dorsum and flanks shagreen with scattered small tubercles (trait more visible in life); low middorsal fold present; dorsolateral folds absent; skin on venter areolate; thoracic and discoidal folds present; cloacal region without ornamentation or evident tubercles.

Ulnar tubercles low (trait more visible in life); outer palmar tubercle prominent, partially divided into a larger (inner) and a smaller (outer) tubercles; thenar tubercle elliptical, the same size with the inner palmar tubercle; subarticular tubercles prominent, round and subconical in section; hyperdistal subarticular tubercles present on all fingers; supernumerary palmar tubercles large, rounded, slightly smaller than subarticular tubercles; fingers bearing lateral fringes; relative length of fingers I < II < IV < III; discs on fingers broadly expanded, rounded; all fingers bearing pads well defined by circumferential grooves (Fig. 11A).

Hindlimbs long (Fig. 10), slender; THL 50% of SVL; TL 52% of SVL; FL 46% of SVL; heel with a slightly larger tubercle (trait more evident in life; Fig. 9); outer edge of tarsus with a row of low tubercles (trait more evident in life); inner edge of tarsus without a fold; inner metatarsal tubercle ovoid, about 5× round outer metatarsal tubercle; subarticular tubercles prominent, round and subconical in section; hyperdistal subarticular tubercles present on all toes; supernumerary plantar tubercles large, rounded, smaller than subarticular tubercles; toes bearing lateral fringes; webbing basal; discs on toes broadly expanded, rounded, smaller than those on fingers; toes with ventral pads well defined by circumferential grooves (Fig. 11B); relative length of toes I < I < III < V < IV; Toe V longer than Toe III on the right foot (tip of Toe III extends beyond proximal edge of penultimate subarticular tubercle on Toe IV, tip of Toe V extends beyond proximal edge and doesn’t reach the distal edge of distal subarticular tubercle on Toe IV); Toe V on the left foot is atypically short, tip of toe barely reaches the proximal edge of penultimate subarticular tubercle on Toe IV.

**Coloration of holotype**. In life (Fig. 9): dorsum light brown; flanks dark brown with white spots of various sizes; dorsal surfaces of hindlimbs dark brown with white spots; the groin and axilla reddish orange (Figs. 9B and 9D); head with faint, light brown, interorbital bar and canthal stripes, and dark brown supratympanic stripes; throat and venter yellowish white to orange with black mottling; iris silver, with a median, broad, red streak and fine black reticulations.

In preservative (Fig. 10): dorsum brown; flanks brown with yellowish white spots; dorsal surfaces of hindlimbs and arms brown with yellowish white spots; the groin and axilla yellowish white; throat and venter yellowish white with brown mottling.

**Measurements of holotype (in mm)**. SVL 29.9; HW 12.8; HL 11.2; IOD 3.8; IND 2.7; EW 3.0; ED 3.9; EN 3.6; TD 1.8; THL 14.9; TL 15.5; FL 13.8; FLL 7.6; HAL 8.7.

**Body mass of holotype**. 2.28 g.

**Variation**. Morphometric variation is shown in Table 1. Coloration does not differ between sexes. Only the female MUTPL 610 (Figs. 12C and 12D) and male MUTPL 612 had white spots on the flanks similar to the holotype. In contrast to the adults, the subadult MUTPL 613 (Figs. 12E and 12F) and the juvenile MUTPL 609 (Figs. 12G and 12H) exhibited mottled green and brown coloration on the dorsum and flanks. Additionally, the venters of these individuals displayed more extensive black mottling compared to those of the adults. In contrast to the silver iris of the holotype and female MUTPL 610 (Fig. 12C), the iris of male MUTPL 616 (Fig. 12A), subadult MUTPL 613 (Fig. 12E), and juvenile MUTPL 609 (Fig. 12G) was light bronze.

**Distribution**. *Pristimantis plateado* is currently known only from a single locality, situated within and immediately adjacent to the Reserva Biológica Cerro Plateado (Fig. 8). However, due to the proximity of the reserve to the Peruvian border, it is likely that *P. plateado* also occurs in adjacent areas of northern Peru. Specimens were encountered at an altitudinal range between 2,195 and 2,536 m in “Montane evergreen forest on sandstone tablelands of the Cordillera del Cóndor” and “Montane evergreen shrubland and grassland of the Cordillera del Cóndor” ecosystems.

**Natural history**. All individuals were encountered at night, either on the ground or perched on leaves or branches at heights ranging from 50 cm to 1.5 m. No calling males were heard. Sympatric frog species include *P. galdi* Jiménez de la Espada, 1870, *P. verrucosus*, *P. melanops* sp. nov., an undescribed *Pristimantis* species and an undescribed *Bolitoglossa* salamander species.

**Conservation status**. *Pristimantis plateado* is known from a single locality, inside and immediately adjacent to a protected area, the Reserva Biológica Cerro Plateado, occupying an estimated area of less than 8 km^2^. Thus, based on the available information (and similarly to *P. verrucosus*), we recommend that *P. plateado* be categorized as Critically Endangered under IUCN criteria B1ab(i,ii,iii,iv)+2ab(i,ii,iii,iv) because: (1) its Extent of Occurrence (EOO) and Area of Occupancy (AOO) are estimated to be less than 8 km^2^; (2) it is known from only one location; and (3) its habitats could be affected in the near future by illegal mining activities.

**Figure 13:**
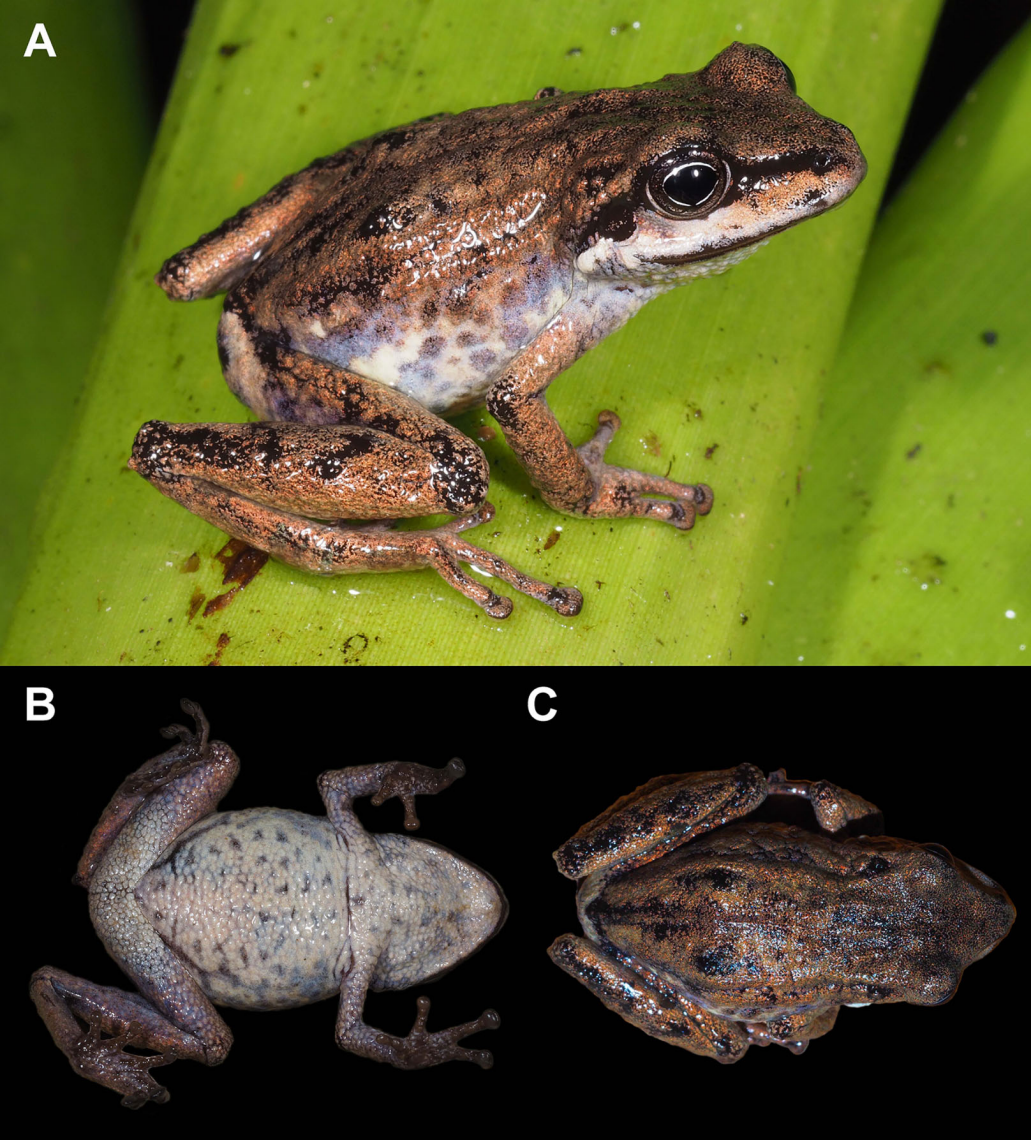
Holotype of *Pristimantis melanops* sp. nov. (MUTPL 599; adult female; SVL 24.3 mm) in life, from Reserva Biológica Cerro Plateado, Zamora Chinchipe Province. (A) dorsolateral view; (B) ventral view; (C) dorsal view.

**Figure 14:**
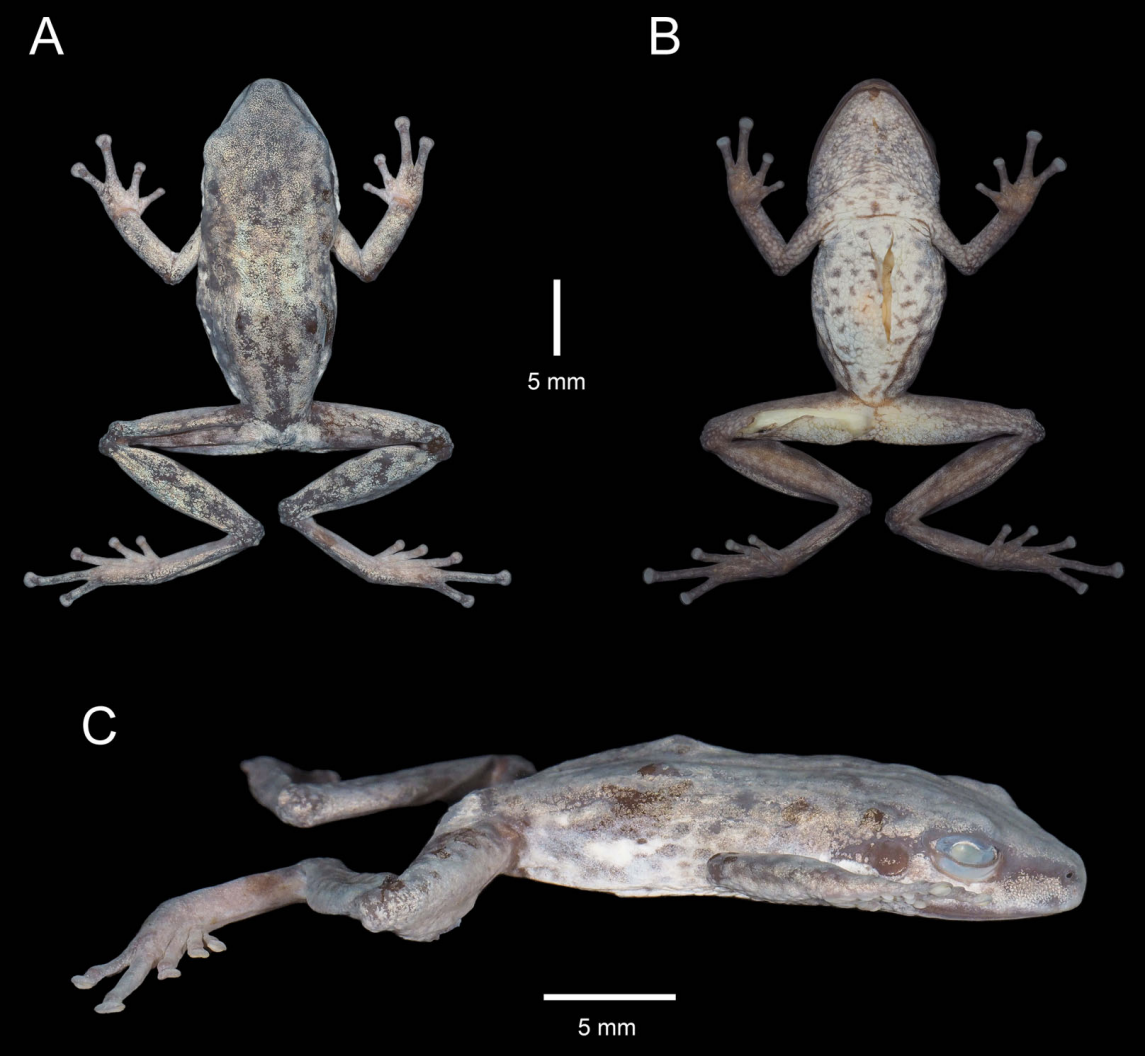
Holotype of *Pristimantis melanops* sp. nov. (MUTPL 599; adult female; SVL 24.3 mm) in preservative, from Reserva Biológica Cerro Plateado, Zamora Chinchipe Province. (A) dorsal view; (B) ventral view; (C) lateral view.

**Figure 15:**
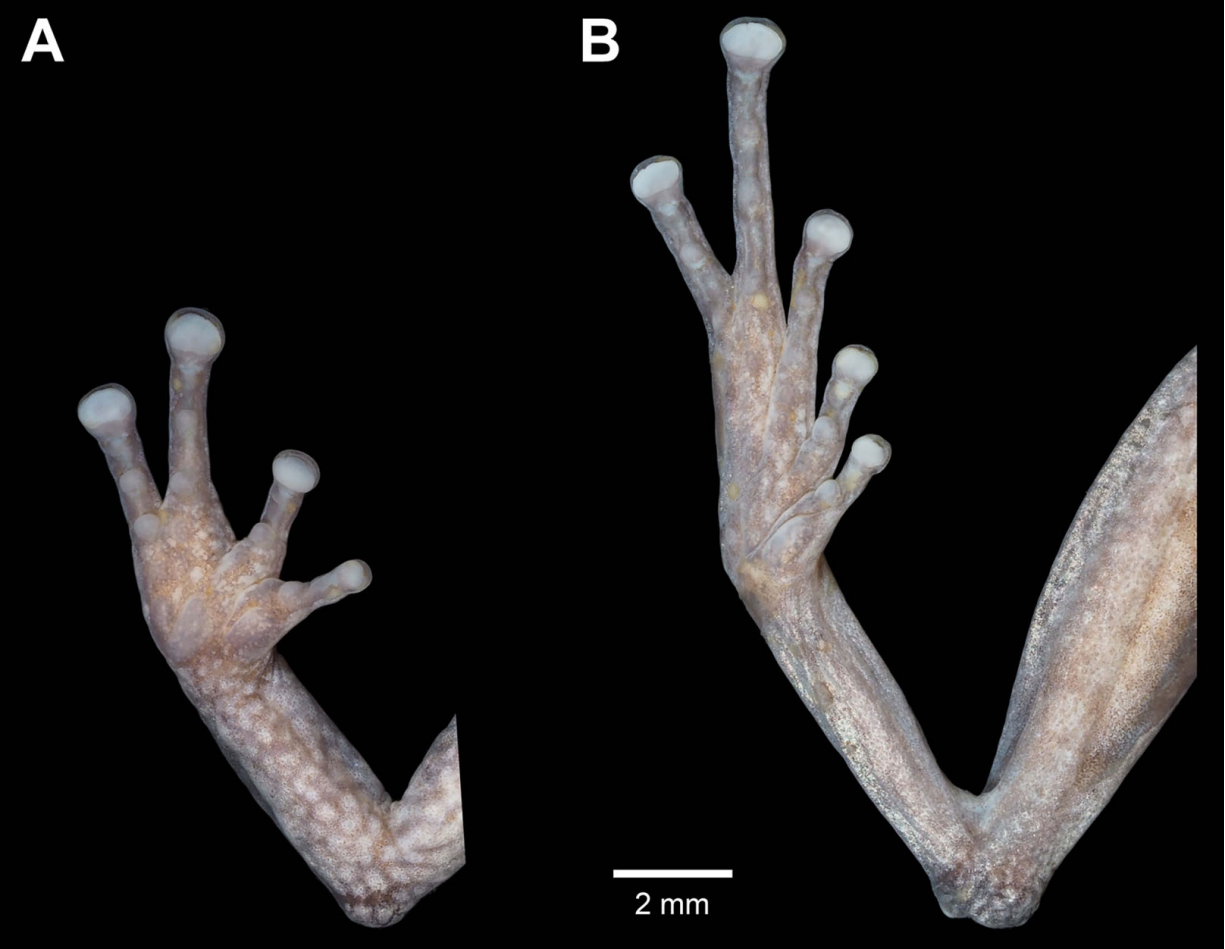
Details of the palmar surface of the hand (A) and plantar surface of the foot (B) of female holotype *Pristimantis melanops* sp. nov. (MUTPL 599; SVL 24.3 mm) in preservative.

**Figure 16:**
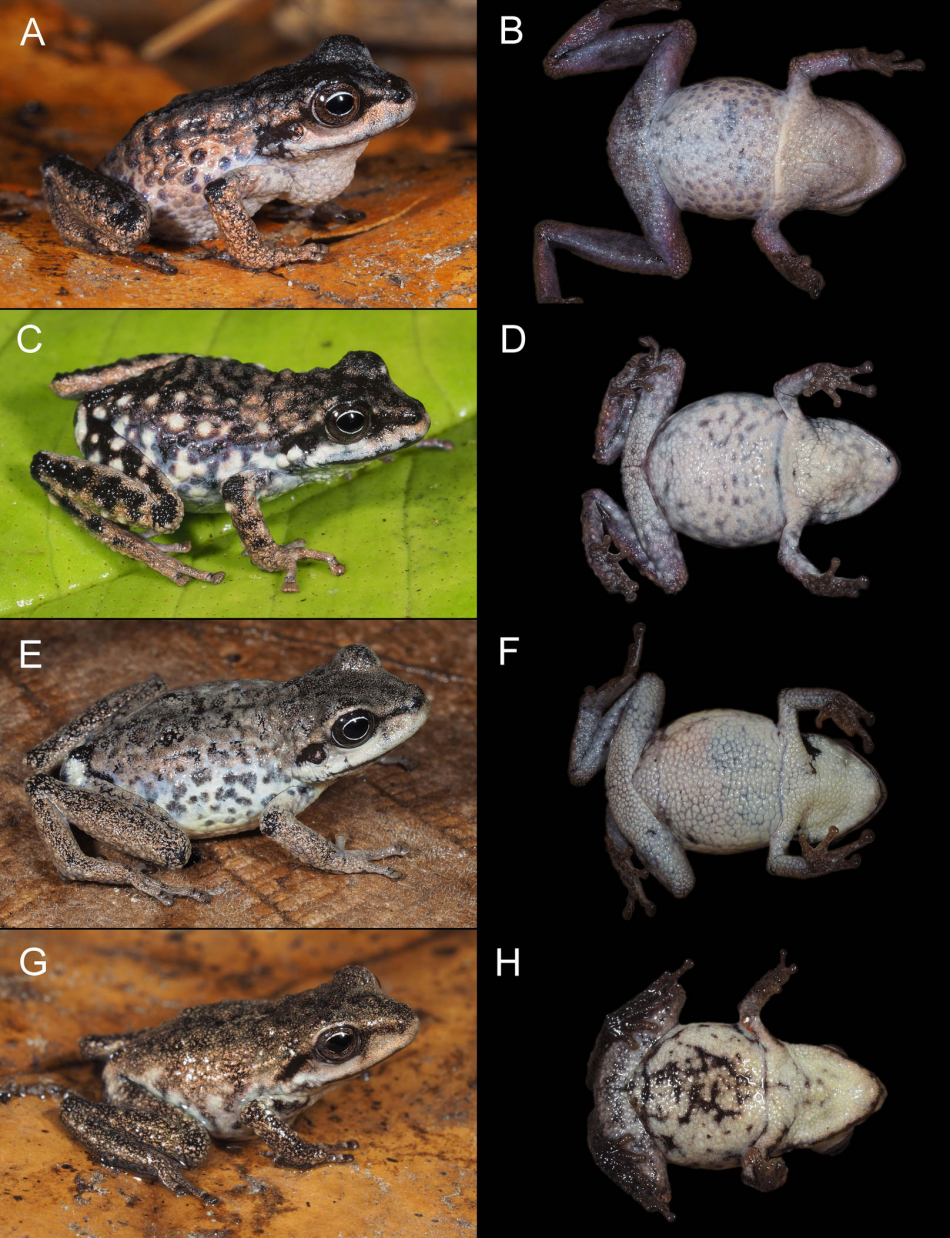
Color variation in life for individuals of *Pristimantis melanops* sp. nov., in dorsolateral and ventral views; all individuals are from Reserva Biológica Cerro Plateado, Zamora Chinchipe Province. (A and B) MUTPL 598, SVL 20.7 mm, adult male paratype, with partially inflated vocal sac; (C and D) MUTPL 2016, SVL 20.8 mm, adult male paratype; (E and F) MUTPL 646, SVL 25.2 mm, adult female paratype; (G and H) MUTPL 645, SVL 14.1 mm, juvenile paratype.

***Pristimantis melanops* sp. nov.**

(Figs. 13, 14, 15 and 16)

ZooBank registration: urn:lsid:zoobank.org:act:957BC889-326E-43B3-9436-2B3F1DDE0158

**Common English name**. Dark-Eyed Rain Frog

**Common Spanish name**. Cutín de ojos oscuros

**Etymology**. The specific epithet *melanops* is derived from the Greek words *melas* (μέλας), meaning “dark” or “black” and *ops* (ὤψ), meaning “eye” or “face”. It refers to the distinctive dark, almost black iris, a unique morphological trait that easily distinguishes this species from its congeners. The name is used as a compound noun in apposition.

**Holotype**. MUTPL 599 (field no. SC 365; Figs. 13, 14 and 15), an adult female from Ecuador, Zamora Chinchipe Province, Reserva Biológica Cerro Plateado (4.6473°S, 78.8777°W; datum WGS84), 2,534 m a.s.l., collected by Santiago Hualpa-Vega and Daniel Hualpa-Vega on 18 January 2019.

**Paratypes.** (Seven: two females, three males, one subadult and one juvenile, all from Zamora Chinchipe Province, Reserva Biológica Cerro Plateado). MUTPL 598 (field no. SC 358), an adult male collected with the holotype; MUTPL 641 (field no. SC 395), an adult male, MUTPL 642 (field no. SC 396), a subadult, MUTPL 645 (field no. SC 399), a juvenile, MUTPL 644 (field no. SC 398) and MUTPL 646 (field no. SC 400), two adult females (4.6476°S, 78.8778°W), 2,535 m a.s.l. collected by Paul Székely, Diana Székely, and Santiago Hualpa-Vega on 27 March 2019; MUTPL 2016 (field no. SC 4144), an adult male (4.6467°S, 78.8770°W), 2,516 m a.s.l., collected by Paul Székely, Diana Székely, and Daniel Hualpa-Vega on 25 September 2025.

**Definition**. We assign this species to *Pristimantis* based on phylogenetic evidence (Fig. 1) and on the general morphological similarity to other members of the genus. *Pristimantis melanops* is a small-sized species (among the *Huicundomantis* subgenus; *sensu* Székely et al., 2026) characterized by the following combination of characters: (1) skin on dorsum shagreen, frequently with large, rounded pustules (trait more evident in life); flanks coarsely pustulated; skin on venter coarsely areolate; discoidal fold present; dorsolateral folds absent; low middorsal fold sometimes present; (2) tympanic annulus evident and tympanic membrane differentiated, its length about 47% of the length of the eye; prominent supratympanic fold present, slightly concealing the upper edge of tympanum; (3) snout subacuminate to rounded in dorsal view, rounded in profile; canthus rostralis straight to slightly concave in dorsal view, rounded in profile; (4) upper eyelid bearing one slightly larger or only small tubercles (trait more evident in life), about 76% IOD in females and 92% IOD in males; cranial crests absent; (5) dentigerous processes of vomers oblique, ovoid, separated medially by distance larger than the width of processes; each process bearing two to four teeth (in females); (6) males with a pigmented subgular vocal sac and vocal slits; (7) fingers relatively short; Finger I shorter than Finger II; discs on fingers slightly expanded, rounded; circumferential grooves present; (8) fingers with lateral fringes; subarticular tubercles prominent; hyperdistal subarticular tubercles present; supernumerary palmar tubercles present; palmar tubercle completely or partially divided into a larger (inner) and a smaller (outer) tubercles; thenar tubercle elliptical, the same size or slightly smaller than the inner palmar tubercle; (9) ulnar tubercles usually present; (10) heel with one slightly larger, rounded tubercle; outer edge of tarsus with a row of low tubercles (trait more evident in life); inner tarsal fold absent, but one or two inner tarsal tubercles are present; (11) inner metatarsal tubercle broadly ovoid, about 4x the size of ovoid outer metatarsal tubercle; subarticular tubercles prominent; hyperdistal subarticular tubercles present; supernumerary plantar tubercles present; (12) toes bearing lateral fringes; webbing basal; Toe V longer than Toe III; discs on toes slightly expanded, rounded, about same size as those on fingers; circumferential grooves present; (13) in life, dorsum, upper parts of flanks, dorsal surfaces of hindlimbs and arms grey, brownish grey, light brown, or black; lower parts of flanks milky white with dark mottling; venter milky white to cream with dark mottling; iris dark, almost black, with white circumpupillary ring; (14) SVL 23.9–25.2 mm in adult females (24.5 ± 0.67, *n* = 3) and 19.5–20.8 mm in adult males (20.3 ± 0.72, *n* = 3).

**Comparison with similar species**. *Pristimantis melanops* exhibits a uniquely distinctive iris coloration that clearly sets it apart from all its congeners. The iris is dark, almost black, with a conspicuous white circumpupillary ring. In reality, the iris is dark bronze, but the dense black reticulations give it an overall blackish appearance (Figs. 13 and 16). We are not aware of any *Pristimantis* species, at least from Ecuador and northern Peru, that exhibit a similar iris coloration.

*Pristimantis melanops* shares with its sister species (*P. multicolor*, *P. percultus*, *P. chomskyi*, *P. andinogigas*, and *P. oculolineatus*; Fig. 1) the characteristic pustulated texture on the flanks (warty or glandular according to other authors: Páez & Ron, 2019; Yánez-Muñoz et al., 2019), but it’s easily distinguished by the smaller size, general blackish dorsal coloration and iris coloration.

**Description of the holotype.** Adult female (MUTPL 599; Figs. 13, 14 and 15), head slightly narrower than body, wider than long, HL 93% of HW, HW 36% of SVL, HL 34% of SVL; snout long (EN 12% of SVL; EN 35% of HL), rounded in dorsal view and rounded in profile; canthus rostralis straight in dorsal view, rounded in profile; loreal region slightly concave; ED larger than EN; nostrils slightly protuberant, oriented posteriorly; lips slightly flared; cranial crests absent; upper eyelid bearing one slightly larger tubercle (trait more visible in life; Fig. 13A); EW 69% of IOD; tympanic annulus evident and tympanic membrane differentiated; prominent supratympanic fold present, slightly concealing the upper margin of the tympanum (Fig. 13A); TD 58% of ED; one (on the right side) and two (on the left side) large, elongated and rounded, white postrictal tubercles; choanae large, oval, partially concealed by palatal shelf of maxillary arch; dentigerous processes of vomers small, smaller than the choanae, oblique, situated posterior and median to choanae, triangular in outline, separated medially by distance much larger than the width of processes, each process bearing two or three teeth; tongue longer than wider, not notched posteriorly, posterior half not adherent to the mouth floor.

Skin on dorsum shagreen; flanks pustulated (Fig. 13A); low middorsal fold present (trait more visible in life; Fig. 13C); dorsolateral folds absent; skin on throat, chest, belly, and posteroventral surfaces of thighs coarsely areolate; thoracic and discoidal folds present; cloacal region bordered ventrally by several small pustules.

Ulnar tubercles present, low (trait more visible in life); outer palmar tubercle completely divided into a larger (inner) and a smaller (outer) tubercles; thenar tubercle elliptical, slightly larger than the inner palmar tubercle; subarticular tubercles prominent, round and rounded in section; hyperdistal subarticular tubercles present in all fingers; supernumerary palmar tubercles large, rounded, slightly smaller than subarticular tubercles; fingers bearing lateral fringes; relative length of fingers I < II < IV < III; discs on fingers slightly expanded, rounded; all fingers bearing pads well defined by circumferential grooves (Fig. 15A).

Hindlimbs relatively short (Fig. 14); THL 47% of SVL; TL 52% of SVL; FL 40% of SVL; heel with one slightly larger, rounded tubercle (Fig. 13A); outer edge of tarsus with a row of low tubercles (trait more evident in life); inner edge of tarsus bearing one tubercle; inner metatarsal tubercle broadly ovoid, about 4× ovoid outer metatarsal tubercle; subarticular tubercles prominent, round and rounded in section; hyperdistal subarticular tubercles present on all toes; supernumerary plantar tubercles rounded, smaller than subarticular tubercles (trait more visible in life); toes bearing lateral fringes; webbing basal; discs on toes slightly expanded, rounded, slightly smaller than those on fingers; toes with ventral pads well defined by circumferential grooves (Fig. 15B); relative length of toes I < II < III < V < IV; Toe V longer than Toe III (tip of Toe III barely extends beyond proximal edge of penultimate subarticular tubercle on Toe IV, tip of Toe V extends beyond proximal edge and doesn’t reach distal edge of distal subarticular tubercle on Toe IV).

**Coloration of holotype.** In life (Fig. 13): dorsum, upper parts of flanks, dorsal surfaces of hindlimbs and arms light brown with some black mottling; lower parts of flanks milky white with brown mottling (some of the pustules are brown); head with black canthal and supratympanic stripes; tympanum black; throat, venter, ventral surfaces of hindlimbs and arms cream with dark brown mottling; iris dark, almost black, with white circumpupillary ring.

In preservative (Fig. 14): dorsum, upper parts of flanks, dorsal surfaces of hindlimbs and arms greyish brown with some dark brown mottling; lower parts of flanks white with brown mottling; head with brown canthal and supratympanic stripes; tympanum brown; throat, venter, ventral surfaces of hindlimbs and arms yellowish white with light brown mottling.

**Measurements of holotype (in mm).** SVL 24.3; HW 8.8; HL 8.2; IOD 3.2; IND 2.1; EW 2.2; ED 3.1; EN 2.9; TD 1.8; THL 11.3; TL 12.7; FL 9.8; FLL 5.9; HAL 6.2.

**Body mass of holotype**. 1.14 g.

**Variation**. Morphometric variation is shown in Table 1. Males exhibit a more pustulate dorsum than the females (Fig. 16). Another notable difference is that, in all three male specimens, the dentigerous processes of vomers were concealed within the buccal mucosa and not evident, unlike in the females. Additionally, the females had more extensive milky white coloration on the flanks, similar to that of the holotype (Fig. 13) and specimen MUTPL 646 (Fig. 16E). Male MUTPL 641 had the dorsum and dorsal surfaces of the limbs almost entirely black, while male MUTPL 2016 (Fig. 16C) displayed numerous white pustules, giving it a distinctly dotted appearance. The subadult MUTPL 642 and juvenile MUTPL 645 (Fig. 16D) showed a generally brownish coloration, and their irises were also less dark compared to those of the adults. In male MUTPL 598 and female MUTPL 644, Toe V on the right foot was much longer than Toe III.

**Distribution**. *Pristimantis melanops* is currently known only from a single locality, situated inside and immediately adjacent to the Reserva Biológica Cerro Plateado (Fig. 8). However, given the proximity of the reserve to the Peruvian border, it is plausible that *P. melanops* also occurs in adjacent areas of northern Peru. Specimens were encountered at an altitudinal range between 2,531 and 2,535 m in a “Montane evergreen shrubland and grassland of the Cordillera del Cóndor” ecosystem.

**Figure 17:**
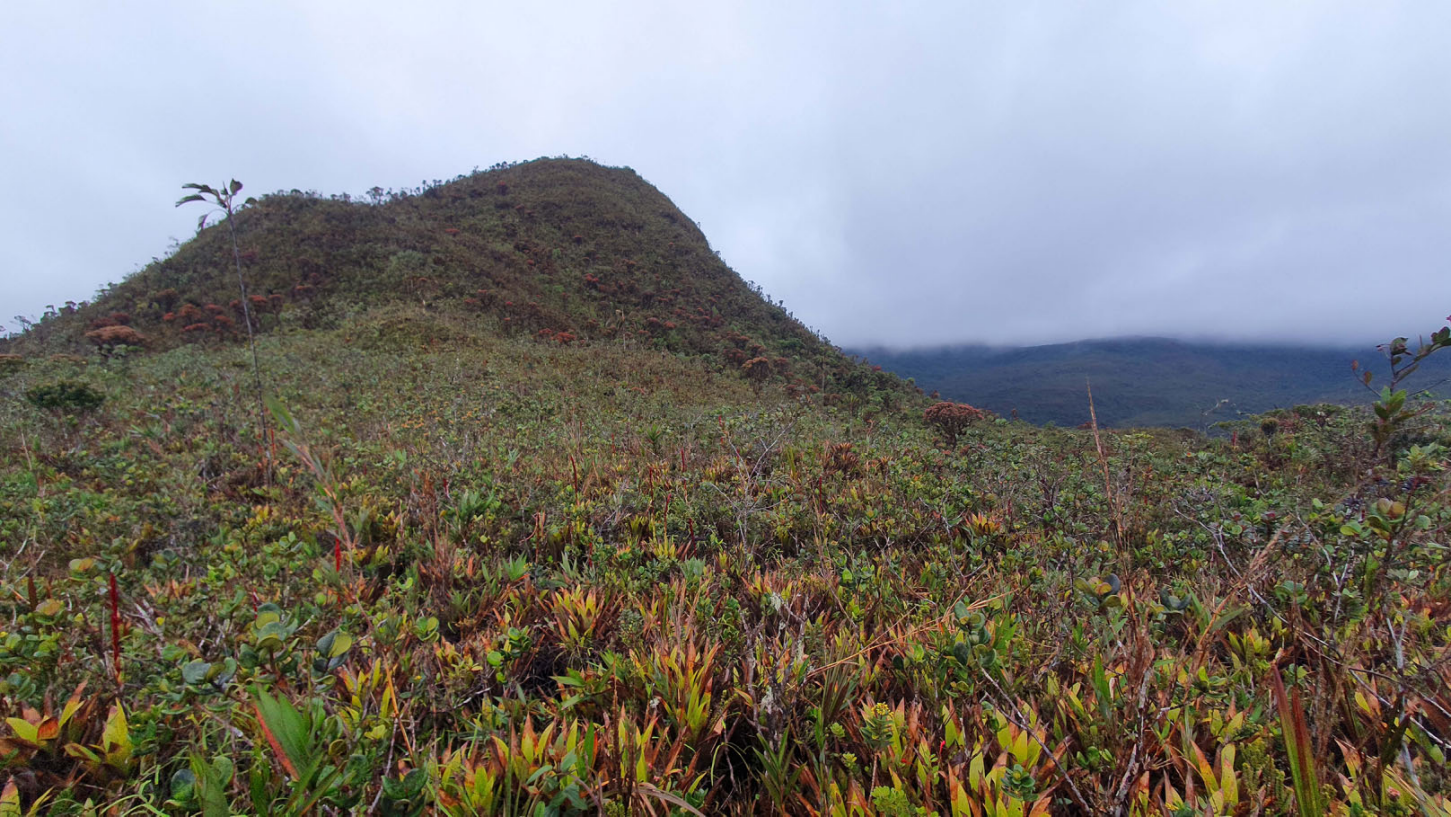
“Montane evergreen shrubland and grassland of the Cordillera del Cóndor” ecosystem in Reserva Biológica Cerro Plateado. This is the typical habitat of *Pristimantis verrucosus* sp. nov. and *P. melanops* sp. nov., characterized by the presence of numerous terrestrial bromeliads.

**Natural history.** Similarly to *P. verrucosus*, we consider this species a bromeliad specialist, as all observed individuals were found inside terrestrial bromeliads (Fig. 17). Specimens were encountered during the night; calling males were heard in March and September. The advertisement call of *P. melanops* appears to be a whistling sound, similar to that of its closest relatives, such as *P. multicolor*, *P. chomskyi*, and *P. andinogigas*. Unfortunately, we were unable to locate calling males and therefore could not obtain recordings of the call. Sympatric frog species include *P. plateado*, *P. verrucosus*, an undescribed *Pristimantis* species and an undescribed *Bolitoglossa* salamander species.

**Conservation status**. *Pristimantis melanops* is known from a single locality, inside and immediately adjacent to a protected area, the Reserva Biológica Cerro Plateado, occupying an estimated area of less than 8 km^2^. Thus, based on the available information (and similarly to *P. verrucosus* and *P. plateado*), we recommend that *P. melanops* be categorized as Critically Endangered under IUCN criteria B1ab(i,ii,iii,iv)+2ab(i,ii,iii,iv) because: (1) its Extent of Occurrence (EOO) and Area of Occupancy (AOO) are estimated to be less than 8 km^2^; (2) it is known from only one location; and (3) its habitats could be affected in the near future by illegal mining activities.

## Discussion

### Systematics

The addition of these three newly described species increases the total number of species in the subgenus *Huicundomantis* to 37, with seven more species remaining undescribed (Székely et al., 2026; Fig. S1). Consistent with Sánchez-Nivicela et al. (2024) and Székely et al. (2026), we recovered the basal terminals within *Huicundomantis* as the clade comprising *P. philipi* and *P. ruidus*, rather than the *P. miktos* group reported by Ortega, Brito & Ron (2022). This difference is most likely due to the distinct gene sampling schemes; however, it is also possible that *P. philipi* and *P. ruidus* belong to an unidentified and unnamed species group. The discovery of additional undescribed species may help clarify the position of this clade within the subgenus.

*Pristimantis verrucosus*, *P. plateado*, and *P. nangaritza* form a strongly supported clade within the *P. cryptomelas* group (Fig. 1). This clade differs markedly from the other major lineage of the group, which includes *P. cryptomelas* and some morphologically similar species. The main distinguishing traits are the absence of the characteristic blackish coloration on the groin and/or the lack of dermal folds or ridges in the occipital/scapular region, except in *P. nangaritza*, which possesses postocular folds. Furthermore, the unique combination of morphological traits in these two new species allows them to be easily distinguished from all other *Pristimantis* species occurring in southern Ecuador and northern Peru.

On the other hand, *P. melanops* belongs to a strongly supported clade within the *P. phoxocephalus* group (Fig. 1), which is characterized by the coarsely pustulated texture on the flanks and a reticulated iris. In *P. melanops*, the reticulations are so dense that they give the iris an overall blackish appearance. Combined with the conspicuous white circumpupillary ring, this iris coloration makes the species unmistakable among all Ecuadorian and northern Peruvian *Pristimantis* species.

### Ecology

The three newly described species exhibit distinct ecological strategies within the unique sandstone plateau ecosystems of the Reserva Biológica Cerro Plateado. *Pristimantis verrucosus* and *P. melanops* were found exclusively in the terrestrial bromeliads located on the crest of a sandstone plateau in the RBCP (Fig. 17). The strict association of these species with bromeliads suggests a high degree of ecological specialization. Their relatively short fingers (compared to other members of the subgenus *Huicundomantis*) with only slightly expanded discs are clear indicators of adaptation to life inside bromeliads (Székely et al., 2020). Similar finger morphology has been reported in other bromeliad-specialist *Pristimantis* species, such as those belonging to the *P. orestes* species group (Brito et al., 2017; Székely et al., 2020).

Bromeliads provide stable microclimates, water reservoirs, and structural complexity, which likely play a critical role in hydration, thermoregulation, and predator avoidance (Ladino et al., 2019). The relatively short fingers and only slightly expanded discs suggest morphological adaptations for moving among the narrow leaf axils of bromeliads. These adaptations may also influence foraging behavior, as bromeliads harbor diverse invertebrate communities that serve as prey.

In contrast, *P. plateado* occupies a broader range of microhabitats, being found on the ground and perched on vegetation up to 1.5 m above the forest floor. This species appears to be a generalist compared to the other two, which may confer it greater resilience to habitat changes.

The ecological specialization of *P. verrucosus* and *P. melanops* increases their vulnerability to environmental disturbances. Because these frogs rely on bromeliads for shelter and possibly reproduction, the loss of these plants could lead to rapid population declines. Furthermore, climate change may exacerbate these threats by altering temperature and humidity regimes essential for bromeliad survival, indirectly impacting these amphibians (Ladino et al., 2019).

Future research should focus on the reproductive ecology of these species, particularly whether they deposit eggs inside bromeliads, as observed in other *Pristimantis* species (Duellman & Lehr, 2009; Rivera-Correa et al., 2021). Understanding their diet and trophic interactions will also provide insights into their ecological roles and the potential cascading effects of bromeliad loss on the broader ecosystem. Given their restricted distributions and high degree of specialization, these species should be considered indicators of ecosystem health and prioritized in conservation planning.

### Conservation

The Reserva Biológica Cerro Plateado is part of Ecuador’s Sistema Nacional de Áreas Protegidas (SNAP), a network of protected areas managed by the Ecuadorian Ministry of Environment (Ministerio del Ambiente del Ecuador, 2018). As a biological reserve, it is a strictly protected area that falls under one of the highest protection categories of the SNAP (Columba Zárate, 2013). Its primary objective is to safeguard its ecosystems with minimal human intervention, while promoting mainly scientific research and conservation activities. Together with the Bosque Protector Alto Nangaritza and Parque Nacional Podocarpus, RBCP forms a vital ecological corridor that ensures connectivity between Andean and Amazonian ecosystems (Jaramillo Mora & Jiménez Ontaneda, 2014).

**Figure 18:**
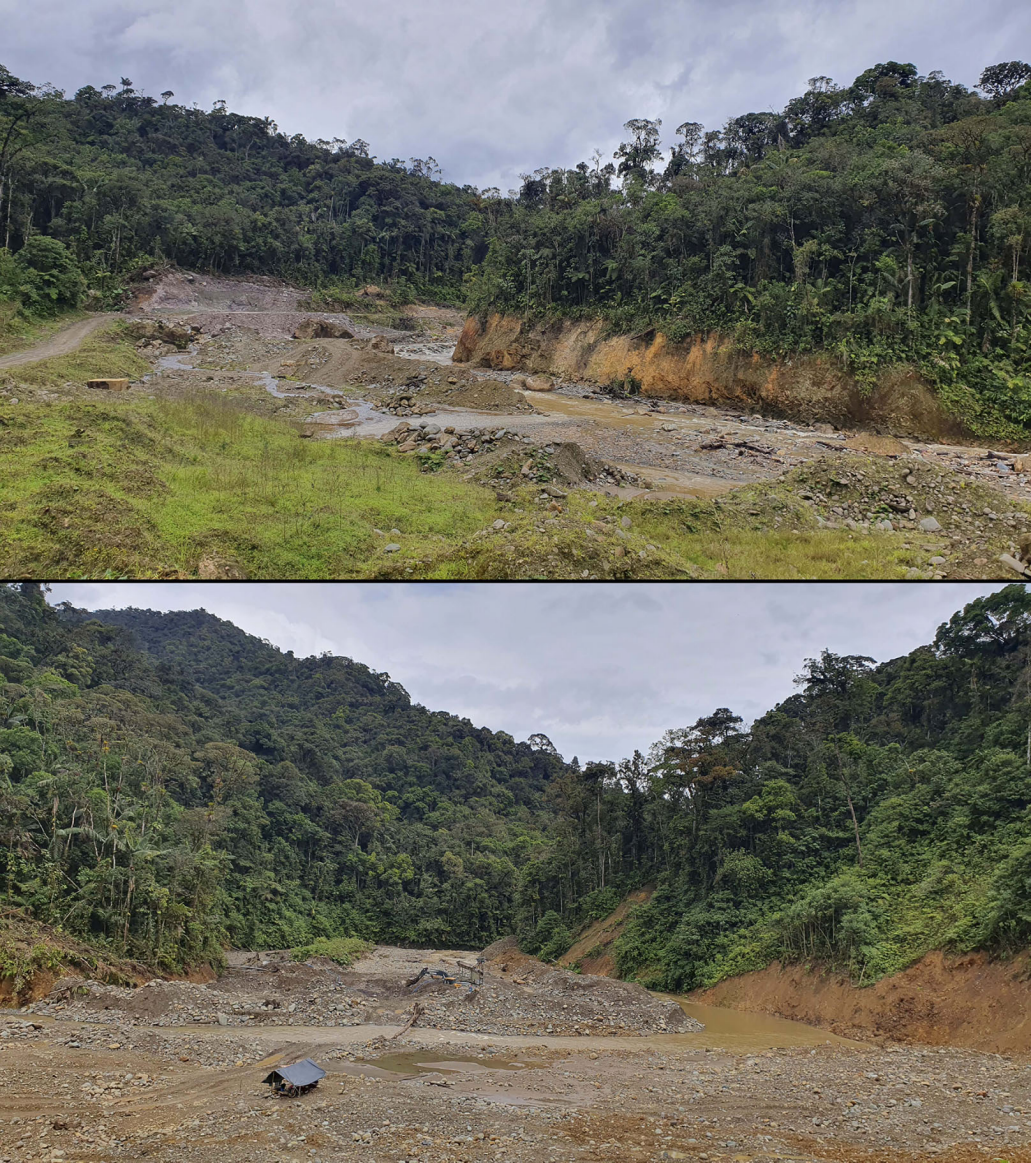
Illegal mining activities along the Panguri River, located within the buffer zone of the Reserva Biológica Cerro Plateado. The images clearly illustrate the extensive environmental degradation, with the river and its bed visibly altered and severely impacted by the mining operations.

Unfortunately, large parts of the Zamora Chinchipe province are severely affected by illegal gold mining, primarily due to weak governmental enforcement. These activities not only degrade ecosystems, but also contribute to corruption and organized crime, undermining governance and threatening local communities (Cogălniceanu & Székely, 2025). Despite their official protected status, areas such as the Bosque Protector Alto Nangaritza and Parque Nacional Podocarpus continue to suffer from illegal mining activities. These operations are well documented (Villa et al., 2022a, 2022b) but often tolerated by the authorities, undermining the conservation efforts. Even with the committed efforts of a small group of rangers striving to protect the area, the RBCP is increasingly threatened, as illegal mining activities have already been reported both within the reserve and in its buffer zone. These operations have led to deforestation and riverbed disruption (Fig. 18), along with mercury, cyanide, and heavy metal pollution, posing serious threats to the region’s biodiversity and ecological integrity.

The reserve safeguards unique sandstone plateau ecosystems that serve as critical refuges for a remarkable diversity of plant and animal species, many of which remain undocumented and are found nowhere else on Earth. This makes the RBCP an invaluable site for conservation. The site remains largely unexplored, offering immense potential for biological research and long-term ecological monitoring. However, without the urgent implementation of effective conservation measures, we risk adding the name of this extraordinary reserve to the growing list of natural areas lost to human greed.

## Conclusions

We present an updated phylogeny of the subgenus *Pristimantis* (*Huicundomantis*) and describe three new species using an integrative taxonomic approach. Two of them, *Pristimantis verrucosus* and *P. plateado*, are placed within the *P. cryptomelas* species group, while the third, *P. melanops*, belongs to the *P. phoxocephalus* group. We propose that all three new species be listed as Critically Endangered due to their restricted distributions and ongoing habitat loss caused by illegal mining activities. Our study highlights the exceptional biodiversity and endemism of the Reserva Biológica Cerro Plateado, a largely unexplored region within the Cordillera del Cóndor.

## Supplemental Information

10.7717/peerj.20930/supp-1 Supplemental Information 1 Maximum likelihood phylogram of the *Pristimantis* (*Huicundomantis*) subgenus based on 2424 base pairs of concatenated mitochondrial DNA from *12S* and *16S*, and nuclear DNA from *RAG-1* gene fragments.

10.7717/peerj.20930/supp-2 Supplemental Information 2 Uncorrected pairwise distances (%), for the mitochondrial gene *16S* fragment in the *Pristimantis* (*Huicundomantis*) subgenus.

10.7717/peerj.20930/supp-3 Supplemental Information 3 12S sequences.

10.7717/peerj.20930/supp-4 Supplemental Information 4 Detailed information regarding the call recordings.

10.7717/peerj.20930/supp-5 Supplemental Information 5 16S sequences.

10.7717/peerj.20930/supp-6 Supplemental Information 6 RAG1 sequences.

10.7717/peerj.20930/supp-7 Supplemental Information 7 Additional specimens examined.

10.7717/peerj.20930/supp-8 Supplemental Information 8 Voucher number, genbank accession numbers, and locality for the *Pristimantis (Huicundomantis)* specimens used in the phylogenetic analysis.

## Additional Information and Declarations

### Field Study Permissions

The following information was supplied relating to field study approvals (*i.e*., approving body and any reference numbers):

Research permits were issued by the Ecuadorian Ministry of Environment (MAE-DNB-CM-2015-0016 and MAATE-ARSFC-2024-1019).

### Data Availability

The following information was supplied regarding data availability:

The aligned and concatenated matrix is available in the Zenodo repository: https://doi.org/10.5281/zenodo.17329629.

The sequences are available at GenBank: PX431658–PX431664, PX431665–PX431671, and PX438739–PX438745.

### New Species Registration

The following information was supplied regarding the registration of a newly described species:

*Pristimantis verrucosus*: urn:lsid:zoobank.org:act:59E22EC4-1087-4CB0-B79F-92028D16D618,

*Pristimantis plateado*: urn:lsid:zoobank.org:act:A15EE2A4-696A-4159-BD8C-A396A840B42A,

*Pristimantis melanops*: urn:lsid:zoobank.org:act:957BC889-326E-43B3-9436-2B3F1DDE0158,

Publication LSID: urn:lsid:zoobank.org:pub:DD6ADBA4-1D9C-4992-B44B-1AF41B091196
