# Supplementary material for: Three new Pristimantis species in the subgenus Huicundomantis (Amphibia: Anura: Strabomantidae) from Reserva Biológica Cerro Plateado, southern Ecuador
Source: PeerJ. 2026 Mar 11;14:e20930. doi: 10.7717/peerj.20930 (PMC12988727; doi:10.7717/peerj.20930)
Supplement: Supplemental Information 1 [file peerj-14-20930-s001.pdf]

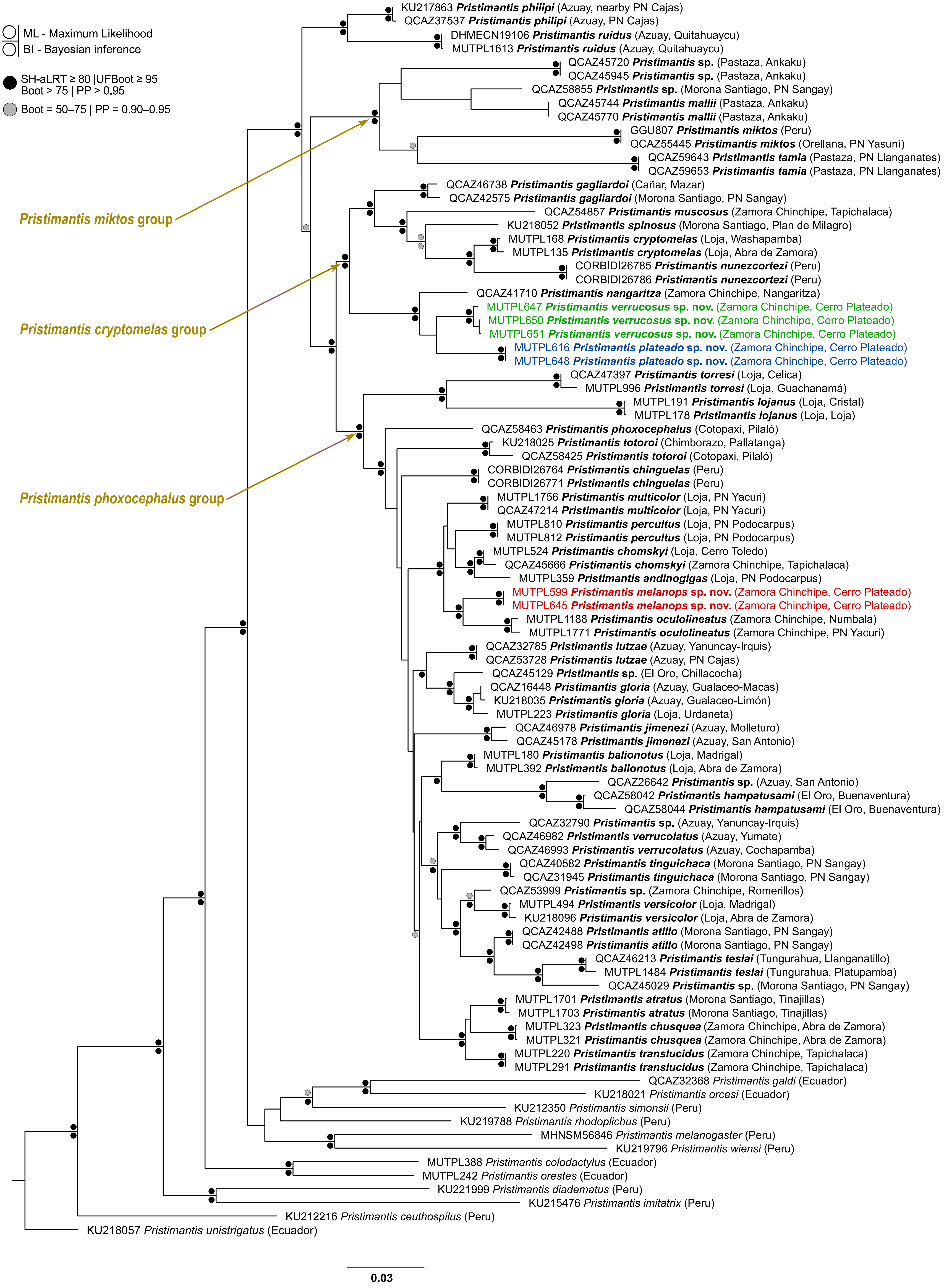

ML - Maximum Likelihood
BI - Bayesian inference
SH-aLRT ≥ 80 |UFBoot ≥ 95
Boot > 75 | PP > 0.95
Boot = 50–75 | PP = 0.90–0.95
Pristimantis miktos group
Pristimantis cryptomelas group
Pristimantis phoxocephalus group
KU217863 Pristimantis philipi (Azuay, nearby PN Cajas)
QCAZ37537 Pristimantis philipi (Azuay, PN Cajas)
DHMECN19106 Pristimantis ruidus (Azuay, Quitahuaycu)
MUTPL1613 Pristimantis ruidus (Azuay, Quitahuaycu)
QCAZ45720 Pristimantis sp. (Pastaza, Ankaku)
QCAZ45945 Pristimantis sp. (Pastaza, Ankaku)
QCAZ58855 Pristimantis sp. (Morona Santiago, PN Sangay)
QCAZ45744 Pristimantis mallii (Pastaza, Ankaku)
QCAZ45770 Pristimantis mallii (Pastaza, Ankaku)
GGU807 Pristimantis miktos (Peru)
QCAZ55445 Pristimantis miktos (Orellana, PN Yasuní)
QCAZ59643 Pristimantis tamia (Pastaza, PN Llanganates)
QCAZ59653 Pristimantis tamia (Pastaza, PN Llanganates)
QCAZ46738 Pristimantis gagliardoi (Cañar, Mazar)
QCAZ42575 Pristimantis gagliardoi (Morona Santiago, PN Sangay)
QCAZ54857 Pristimantis muscosus (Zamora Chinchipe, Tapichalaca)
KU218052 Pristimantis spinosus (Morona Santiago, Plan de Milagro)
MUTPL168 Pristimantis cryptomelas (Loja, Washapamba)
MUTPL135 Pristimantis cryptomelas (Loja, Abra de Zamora)
CORBIDI26785 Pristimantis nunezcortezi (Peru)
CORBIDI26786 Pristimantis nunezcortezi (Peru)
QCAZ41710 Pristimantis nangaritza (Zamora Chinchipe, Nangaritza)
MUTPL647 Pristimantis verrucosus sp. nov. (Zamora Chinchipe, Cerro Plateado)
MUTPL650 Pristimantis verrucosus sp. nov. (Zamora Chinchipe, Cerro Plateado)
MUTPL651 Pristimantis verrucosus sp. nov. (Zamora Chinchipe, Cerro Plateado)
MUTPL616 Pristimantis plateado sp. nov. (Zamora Chinchipe, Cerro Plateado)
MUTPL648 Pristimantis plateado sp. nov. (Zamora Chinchipe, Cerro Plateado)
QCAZ47397 Pristimantis torresi (Loja, Celica)
MUTPL996 Pristimantis torresi (Loja, Guachanamá)
MUTPL191 Pristimantis lojanus (Loja, Cristal)
MUTPL178 Pristimantis lojanus (Loja, Loja)
QCAZ58463 Pristimantis phoxocephalus (Cotopaxi, Pilaló)
KU218025 Pristimantis totoroi (Chimborazo, Pallatanga)
QCAZ58425 Pristimantis totoroi (Cotopaxi, Pilaló)
CORBIDI26764 Pristimantis chinguelas (Peru)
CORBIDI26771 Pristimantis chinguelas (Peru)
MUTPL1756 Pristimantis multicolor (Loja, PN Yacuri)
QCAZ47214 Pristimantis multicolor (Loja, PN Yacuri)
MUTPL810 Pristimantis percultus (Loja, PN Podocarpus)
MUTPL812 Pristimantis percultus (Loja, PN Podocarpus)
MUTPL524 Pristimantis chomskyi (Loja, Cerro Toledo)
QCAZ45666 Pristimantis chomskyi (Zamora Chinchipe, Tapichalaca)
MUTPL359 Pristimantis andinogigas (Loja, PN Podocarpus)
MUTPL599 Pristimantis melanops sp. nov. (Zamora Chinchipe, Cerro Plateado)
MUTPL645 Pristimantis melanops sp. nov. (Zamora Chinchipe, Cerro Plateado)
MUTPL1188 Pristimantis oculolineatus (Zamora Chinchipe, Numbala)
MUTPL1771 Pristimantis oculolineatus (Zamora Chinchipe, PN Yacuri)
QCAZ32785 Pristimantis lutzae (Azuay, Yanuncay-Irquis)
QCAZ53728 Pristimantis lutzae (Azuay, PN Cajas)
QCAZ45129 Pristimantis sp. (El Oro, Chillacocha)
QCAZ16448 Pristimantis gloria (Azuay, Gualaceo-Macas)
KU218035 Pristimantis gloria (Azuay, Gualaceo-Limón)
MUTPL223 Pristimantis gloria (Loja, Urdaneta)
QCAZ46978 Pristimantis jimenezi (Azuay, Molleturo)
QCAZ45178 Pristimantis jimenezi (Azuay, San Antonio)
MUTPL180 Pristimantis balionotus (Loja, Madrigal)
MUTPL392 Pristimantis balionotus (Loja, Abra de Zamora)
QCAZ26642 Pristimantis sp. (Azuay, San Antonio)
QCAZ58042 Pristimantis hampatusami (El Oro, Buenaventura)
QCAZ58044 Pristimantis hampatusami (El Oro, Buenaventura)
QCAZ32790 Pristimantis sp. (Azuay, Yanuncay-Irquis)
QCAZ46982 Pristimantis verrucolatus (Azuay, Yumate)
QCAZ46993 Pristimantis verrucolatus (Azuay, Cochapamba)
QCAZ40582 Pristimantis tinguichaca (Morona Santiago, PN Sangay)
QCAZ31945 Pristimantis tinguichaca (Morona Santiago, PN Sangay)
QCAZ53999 Pristimantis sp. (Zamora Chinchipe, Romerillos)
MUTPL494 Pristimantis versicolor (Loja, Madrigal)
KU218096 Pristimantis versicolor (Loja, Abra de Zamora)
QCAZ42488 Pristimantis atillo (Morona Santiago, PN Sangay)
QCAZ42498 Pristimantis atillo (Morona Santiago, PN Sangay)
QCAZ46213 Pristimantis teslai (Tungurahua, Llanganatillo)
MUTPL1484 Pristimantis teslai (Tungurahua, Platupamba)
QCAZ45029 Pristimantis sp. (Morona Santiago, PN Sangay)
MUTPL1701 Pristimantis atratus (Morona Santiago, Tinajillas)
MUTPL1703 Pristimantis atratus (Morona Santiago, Tinajillas)
MUTPL323 Pristimantis chusquea (Zamora Chinchipe, Abra de Zamora)
MUTPL321 Pristimantis chusquea (Zamora Chinchipe, Abra de Zamora)
MUTPL220 Pristimantis translucidus (Zamora Chinchipe, Tapichalaca)
MUTPL291 Pristimantis translucidus (Zamora Chinchipe, Tapichalaca)
QCAZ32368 Pristimantis galdi (Ecuador)
KU218021 Pristimantis orcesi (Ecuador)
KU212350 Pristimantis simonsii (Peru)
KU219788 Pristimantis rhodoplichus (Peru)
MHNSM56846 Pristimantis melanogaster (Peru)
KU219796 Pristimantis wiensi (Peru)
MUTPL388 Pristimantis colodactylus (Ecuador)
MUTPL242 Pristimantis orestes (Ecuador)
KU221999 Pristimantis diadematus (Peru)
KU215476 Pristimantis imitatrix (Peru)
KU212216 Pristimantis ceuthospilus (Peru)
KU218057 Pristimantis unistrigatus (Ecuador)
0.03
