# Supplementary material for: Three new Pristimantis species in the subgenus Huicundomantis (Amphibia: Anura: Strabomantidae) from Reserva Biológica Cerro Plateado, southern Ecuador
Source: PeerJ. 2026 Mar 11;14:e20930. doi: 10.7717/peerj.20930 (PMC12988727; doi:10.7717/peerj.20930)
Supplement: Supplemental Information 7 [file peerj-14-20930-s007.docx]

**APPENDIX 1. ADDITIONAL SPECIMENS EXAMINED**

*Pristimantis andinogigas* (1)*.* ECUADOR: LOJA PROVINCE, Parque Nacional Podocarpus - Cajanuma (MUTPL 359).

*Pristimantis balionotus* (14)*.* ECUADOR: LOJA PROVINCE, Abra de Zamora (MUTPL 292, 297, 391, 392, 677); Reserva Madrigal del Podocarpus (MUTPL 180, 487, 489–491); ZAMORA CHINCHIPE PROVINCE, Estación Científica San Francisco (MUTPL 1673, 1674, 1676, 1678)

*Pristimantis chomskyi* (10)*.* ECUADOR: LOJA PROVINCE, Parque Nacional Podocarpus - Cerro Toledo (MUTPL 113–116, 121, 524–526, 816); ZAMORA CHINCHIPE PROVINCE, Reserva Tapichalaca (MUTPL 1783).

*Pristimantis cryptomelas* (16)*.* ECUADOR: LOJA PROVINCE, Abra de Zamora (MUTPL 135, 470, 471); Bosque Protector Washapamba (MUTPL 167–171); Loja, Huacapamba (MUTPL 380, 381, 383, 385); Parque Nacional Podocarpus - Cajanuma (MUTPL 493); San Lucas (MUTPL 1121); ZAMORA CHINCHIPE PROVINCE, Reserva Numbala (MUTPL 1174); Parque Nacional Yacuri (MUTPL 1774).

*Pristimantis gloria* (56)*.* ECUADOR: AZUAY PROVINCE, Vía Gualaceo - Plan de Milagro, Loma de la Virgen (1623); LOJA PROVINCE, Vía Urdaneta-Tutupali (MUTPL 222–238, 250); MORONA SANTIAGO PROVINCE, Bosque Protector Jima (MUTPL 39–72); Área Ecológica de Conservación Municipal Tinajillas Río Gualaceño (MUTPL 1624); ZAMORA CHINCHIPE PROVINCE, Belen, Bosque Protector Shincata (MUTPL 801).

*Pristimantis lojanus* (32). ECUADOR: LOJA PROVINCE, Cristal, Reserva El Cristal (MUTPL 191, 192, 280); Loja (MUTPL 14–23, 108, 176–179, 628, 935, 936, 1030, 1076); Parque Nacional Podocarpus - Cajanuma (MUTPL 923); San Lucas (MUTPL 704, 1026).

*Pristimantis multicolor* (1). ECUADOR: LOJA PROVINCE, Parque Nacional Yacuri (MUTPL 1756).

*Pristimantis muscosus* (4). ECUADOR: ZAMORA CHINCHIPE PROVINCE, La Canela (MUTPL 1553); Reserva Biológica Cerro Plateado (MUTPL 619, 637); Reserva Tapichalaca (MUTPL 741).

*Pristimantis percultus* (3)*.* ECUADOR: LOJA PROVINCE, Parque Nacional Podocarpus - Cajanuma (MUTPL 810–812).

*Pristimantis ruidus* (1). ECUADOR: AZUAY PROVINCE, Reserva Quitahuaycu (MUTPL 1613).

*Pristimantis spinosus* (3). ECUADOR: MORONA SANTIAGO PROVINCE, Área Ecológica de Conservación Municipal Tinajillas Río Gualaceño (MUTPL 1638, 1639, 1699).

*Pristimantis teslai* (3). ECUADOR: TUNGURAHUA PROVINCE, El Triunfo (MUTPL 1425); Patate (MUTPL 1484, 1489).

*Pristimantis torresi* (5)*.* ECUADOR: EL ORO PROVINCE, Nudillo (MUTPL 1847, 1876); LOJA PROVINCE, Guachanamá, El Apretadero (MUTPL 996–998).

*Pristimantis versicolor* (23)*.* ECUADOR: LOJA PROVINCE, Abra de Zamora (MUTPL 112, 293, 294, 313, 389, 390, 497); Loja, Huacapamba (MUTPL 806); Parque Nacional Podocarpus - Cajanuma (MUTPL 910); Parque Nacional Podocarpus - El Palto (MUTPL 991); Ramos Urcu (MUTPL 719); Reserva Madrigal del Podocarpus (MUTPL 494); ZAMORA CHINCHIPE PROVINCE, Abra de Zamora (MUTPL 1522); Estación Científica San Francisco (MUTPL 1615, 1842); La Canela (MUTPL 1563, 1566); Reserva Biológica Cerro Plateado (MUTPL 653); Reserva Numbala (MUTPL 1175); Reserva Tapichalaca (MUTPL 738–740, 1787).
